# Chromosomal evolution in the plant family Solanaceae

**DOI:** 10.1186/1471-2164-11-182

**Published:** 2010-03-17

**Authors:** Feinan Wu, Steven D Tanksley

**Affiliations:** 1 Department of Plant Breeding and Genetics, Cornell University, Ithaca, NY 14853, USA; 2 Department of Plant Biology, Cornell University, Ithaca, NY 14853, USA

## Abstract

**Background:**

Over the past decades, extensive comparative mapping research has been performed in the plant family Solanaceae. The recent identification of a large set of single-copy conserved orthologous (COSII) markers has greatly accelerated comparative mapping studies among major solanaceous species including tomato, potato, eggplant, pepper and diploid *Nicotiana *species (as well as tetraploid tobacco). The large amount of comparative data now available for these species provides the opportunity to describe the overall patterns of chromosomal evolution in this important plant family. The results of this investigation are described herein.

**Results:**

We combined data from multiple COSII studies, and other comparative mapping studies performed in tomato, potato, eggplant, pepper and diploid *Nicotiana *species, to deduce the features and outcomes of chromosomal evolution in the Solanaceae over the past 30 million years. This includes estimating the rates and timing of chromosomal changes (inversions and translocations) as well as deducing the age of ancestral progenitor species and predicting their genome configurations.

**Conclusions:**

The Solanaceae has experienced chromosomal changes at a modest rate compared with other families and the rates are likely conserved across different lineages of the family. Chromosomal inversions occur at a consistently higher rate than do translocations. Further, we find evidences for non-random positioning of the chromosomal rearrangement breakpoints. This finding is consistent with the similar finding in mammals, where hot spots for chromosomal breakages have apparently played a significant role in shaping genome evolution. Finally, by utilizing multiple genome comparisons we were able to reconstruct the most likely genome configuration for a number of now-extinct progenitor species that gave rise to the extant solanaceous species used in this research. The results from this study provide the first broad overview of chromosomal evolution in the family Solanaceae, and one of the most detailed thus far for any family of plants.

## Background

The Solanaceae is a large plant family comprised of over 3000 species including many important crops such as tomato, potato, eggplant, and pepper. It represents a group of dicotyledonous plants in the Euasterid clade, which is divergent from the model plant Arabidopsis [1,2]. It is the third economically important plant family and ranks the first in terms of vegetable crops. The related family Rubiaceae contains coffee: one of the most valuable agricultural commodities in the international trade. Unlike some other plant families (e.g. Poaceae and Brassicaceae), the Solanaceae and the Rubiaceae have evolved largely in the absence of polyploidization [3]. For instance, tomato, eggplant and pepper have the same basic chromosome number (2n = 24) and share similar chromosome architecture with pericentric heterochromatin and more distal euchromatic arms. Cultivated potato and tobacco are tetraploid but the events occurred relatively recently and many diploid species can still be found in the wild. Coffee also has a similar chromosome architecture and DNA content, and has a chromosome number of 2n = 22. As a result, the Solanaceae and related taxa present a unique opportunity to study genome/gene evolution in the absence of recent polyploidization events.

In the past two decades, extensive pairwise comparative mapping studies have been performed for several major solanaceous crops relative to tomato [4-8]. However, no comparisons of multiple species have been conducted to decipher the overall patterns of chromosomal evolution in this family. Recently, Wu et al [3] developed a set of 2869 single-copy conserved ortholog set (COSII) markers of which 877 have been mapped in the tomato genome http://sgn.cornell.edu/cview/map.pl?map_id=9&show_offsets=1&show_ruler=1. Subsequently, COSII genetic maps have been constructed for eggplant, pepper and two diploid *Nicotiana *species, and pairwise comparisons have been reported between each species and tomato [9-11]. Potato, a close taxonomic relative of tomato, was also included in this series of comparisons [8,12]. These previous comparative mapping studies have established the syntenic relationships between the 12 tomato chromosomes (T1-12) and those of potato (Pt1-12), eggplant (E1-12), pepper (P1-12) and *Nicotiana *(N1-12). The *Nicotiana *map used in this work is a deduced map for the diploid ancestor of *N. tomentosiformis *and *N. acuminata *(referred to *Nicotiana *hereafter) based on comparisons of these two species and tomato [11]. The deduced map of the ancestral *Nicotiana *species excludes inversions that occurred subsequent to the divergence of *N. tomentosiformis *and *N. acuminata*.

Previous comparative mapping studies across solanaceous species have revealed that inversions have been the most common cause of genome rearrangements--with translocations occurring at a lower frequency. Tomato and potato differ by 6 inversions [8,12]. Tomato and eggplant differ by 24 inversions and 5 translocations [10]. Tomato and pepper differ by 19 inversions and 6 translocations [9]. Tomato and *Nicotiana *differ by a minimum of 10 inversions and 11 translocations, although the number of inversions is likely underestimated due to the large evolutionary distance between tomato and *Nicotiana *[11]. In the current study we performed multiple-species comparisons in order to estimate the timing of these chromosomal rearrangement events, deduce the genome arrangement of the most recent common ancestors (MRCAs) of these species, and compare the rates of chromosomal evolution across different lineages. We also compared these results for the Solanaceae with similar studies in other plant and animal families.

## Methods

### Estimating divergence time for species in the family Solanaceae

**Figure 1:**
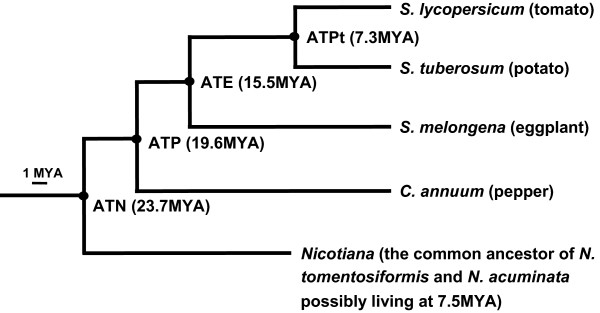
**Phylogenetic relationships and estimated divergence time of selected solanaceous species**. The phylogenetic tree with molecular dating is based on Additional File 1. The external node *Nicotiana *represents the most recent common ancestor of *N. tomentosiformis *and *N. acuminata*, an extinct species possibly living at 7.5MYA [11]. Estimated age of the 4 ancestral species (ATPt, ATE, ATP and ATN) is placed next to the corresponding interval node (highlighted with a black dot).

Wu et al [3] published a phylogenetic tree of solanaceous species with strong statistical support (>97% bootstrap values for all branches) based on concatenated exons of 10 COSII markers. Using the same dataset and the same method, we reconstructed a phylogenetic tree rooted with coffee (The previous root Arabidopsis was removed because of its large phylogenetic distance to the other species). Subsequently, we ran the program r8s [13] to estimate the age of internal nodes by the non-parametric rate smoothing (NPRS) method, which does not rely on the assumption of a molecular clock [14]. The calibration point was tomato-coffee split at 83 ~ 89 million years ago (MYA), and 86MYA was used for calculation [15]. Additional File 1 presents the complete result from this analysis and Figure 1 only includes the species used for the current study.

### Comparing the maps of multiple solanaceous species

**Figure 2:**
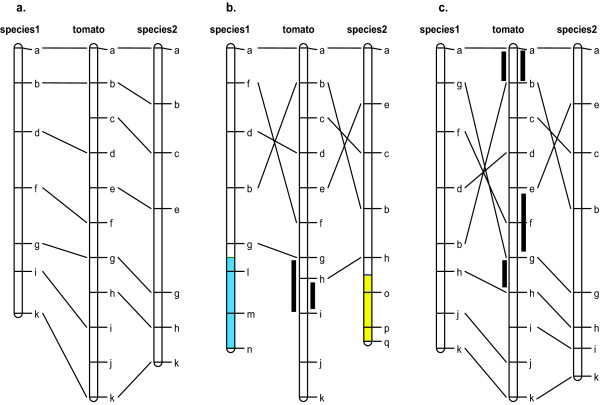
**Comparing maps of multiple species using the tomato genetic map as common reference**. (a) Species 1 and 2 both share the same gene order with tomato. (b) Species 1 and 2 both have an inversion and a translocation relative to tomato. Markers in blue and yellow segments come from two other tomato chromosomes. Breakpoint region (indicated by a black bar) of the translocation between species 1 and tomato is the interval of markers "g" and "i", and similarly that between species 2 and tomato is the interval of markers "h" and "i". (c) Species 1 and 2 each have an independent inversion relative to tomato. Breakpoint regions are indicated by black bars next to the tomato map.

As a starting point, we utilized previously published comparisons of tomato and each of the other solanaceous species included in this current study--potato [8,12], eggplant [10], pepper [9] and *Nicotiana *[11]. The tomato genetic map has the largest number of markers, including all the orthologous markers employed in these pairwise comparisons [3]; however, different subsets (although they may share a relatively small number of common markers) were mapped in each of the other species being compared--making it difficult to perform direct comparisons between non-tomato species (potato, eggplant, pepper and *Nicotiana*). Using the principle of parsimony, we applied the following rules to overcome these difficulties. For any genomic region being compared, if a pair of non-tomato species was determined to have the same gene order as tomato, we considered that the pair of non-tomato species also shared the same gene order with respect to each other in this particular region (Figure 2a). If on the other hand, each of the non-tomato species differed from tomato by an inversion corresponding to the same region of the tomato genome, we considered that the pair of non-tomato species had the same gene order and the inversion was derived along the tomato lineage (Figure 2b). However, in the situation shown in Figure 2c, we considered that species 1 and 2 each had an independent inversion relative to tomato even though the two inverted regions overlapped with each other. Lower breakpoint of the inversion between species 1 and tomato was located between markers "g" and "h" on the tomato map while that between species 2 and tomato was between markers "e" and "g", in other words, the two breakpoints were different and therefore they must have been resulted from two independent events. For translocations (Figure 2b), if the breakpoint region of the translocation between species 1 and tomato (between markers "g" and "i") overlapped with that between species 2 and tomato (between markers "h" and "i"), we considered that the two species possibly shared the same breakpoint and presumably the breakpoint region could be narrowed down to the overlapping region.

### Deducing the genome arrangement of ancestral species

We employed the principle of parsimony to deduce the genome arrangement of three ancestral species--ATPt, ATE and ATP (see Figure 1 for definition). If tomato and potato shared the same genome arrangement, they both likely preserved the arrangement of ATPt. If tomato and potato differed from each other, the species, which shared the same arrangement with any of the more distant species--eggplant, pepper and *Nicotiana*, would be considered to preserve the arrangement of ATPt while the other one had the derived condition. Using the same method, we compared ATPt and eggplant (together with the more distant species pepper and *Nicotiana*) to decide which one or both preserved the arrangement of ATE, and again we compared ATE and pepper (together with the more distant species *Nicotiana*) to decide which one or both preserved the arrangement of ATP.

## Results

### Phylogenetic relationships of the studied solanaceous species and the estimated age of their most recent common ancestors (MRCAs)

The phylogenetic relationships of species in the family Solanaceae have been studied using both plastid and nuclear sequences [3,16-18]. Among the solanaceous species included in the current study, tomato, potato and eggplant belong to the genus *Solanum*, with tomato and potato closer to each other than to eggplant. Pepper is a member of the genus *Capsicum*. *Nicotiana *is the most distant and basal to the other two genera (Figure 1). Molecular dating (Figure 1; see Methods) suggested 7.3MYA for the age of the MRCA of tomato and potato (referred to as ATPt hereafter), 15.5MYA for that of the MRCA of tomato, potato and eggplant (ATE), 19.6MYA for that of the MRCA of tomato, potato, eggplant and pepper (ATP), and 23.7MYA for that of the MRCA of tomato, potato, eggplant, pepper and *Nicotiana *(ATN). These estimates are similar to those reported from recent studies [15,19,20].

### Inferring the arrangement of MRCA genomes

**Figure 3:**
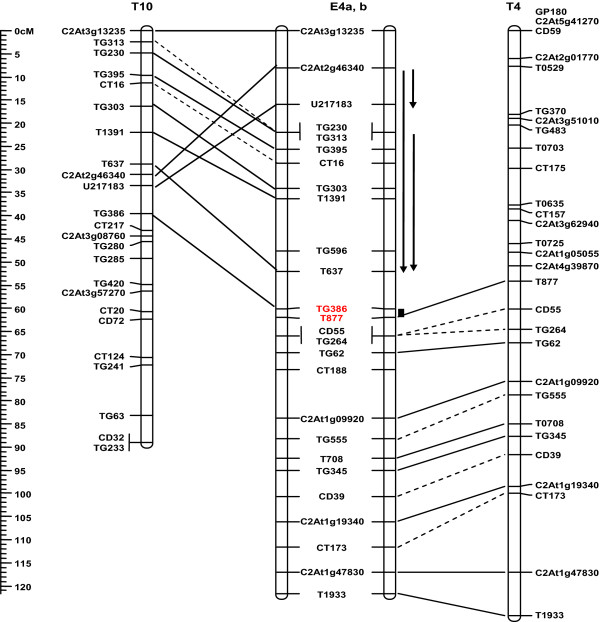
**An example of chromosomal rearrangements between the genomes of eggplant and tomato**. The eggplant chromosome E4 combines two segments (E4a and E4b) orthologous to tomato T4 and T10 respectively, indicating a translocation between the two genomes. The breakpoint is located between markers TG386 and T677 (highlighted in red), and the region is indicated by a black bar beside E4. Orthologous marker pairs are connected by lines. A dash line indicates a marker of low mapping confidence on either or both maps that is not used for deduction of inversions. Vertical arrows beside E4 depict inversions in E4 with respect to T10.

The tomato genetic map was used as standard in this work since it shared the most orthologous markers with the maps of potato, eggplant, pepper and *Nicotiana*. After these species diverged from their common ancestors with tomato, numerous translocations have reshuffled the ancestral 12 chromosomes so that there is not always a 1:1 correspondence for the orthologous chromosomes among these species (the only exception is that the tomato and potato genomes do not differ by any translocations). To facilitate the following analyses, we divided the 12 chromosomes in eggplant, pepper and *Nicotiana *into segments according to their translocations relative to tomato (Additional File 2). For example, eggplant E4 combined segments from tomato T10 and T4 respectively and thus was divided into segments E4a and E4b (Figure 3).

**Figure 4:**
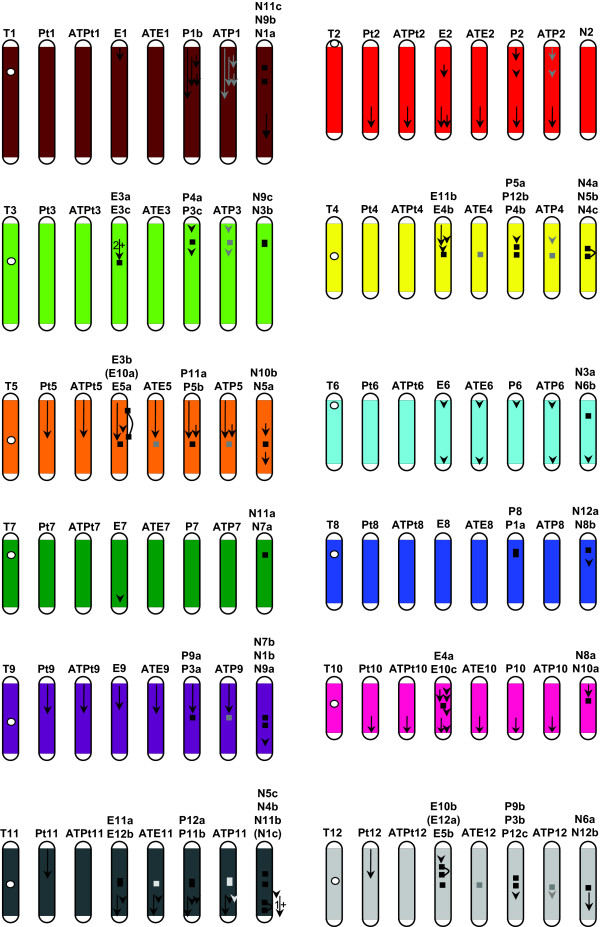
**Comparative maps of several solanaceous species and the deduced genome arrangement of MRCAs**. T1-12, Pt1-12, E1-12, P1-12 and N1-12 represent 12 chromosomes of tomato, potato, eggplant, pepper and *Nicotiana *genomes respectively. Designation of chromosome segments (a-c) is detailed in Additional File 2. ATPt1-12, ATE1-12 and ATP1-12 represent 12 chromosomes of ATPt, ATE and ATP genomes. White dots indicate the approximate centromere location of the tomato chromosomes. Maps of the other species are depicted in a comparative way to the tomato map as follows. A black arrow indicates an inversion relative to tomato (a grey arrow for an uncertain inversion). A black bar indicates the breakpoint region of a translocation relative to tomato (a grey bar for an uncertain translocation). Two black bars connected by a curve indicate that the segment in between is excised in a translocation while the remained parts stay together, e.g. E10a is embedded in E3b. "1+" (or "2+") on a single arrow indicates that the region has experienced at least one (or two) inversions but the exact number remains to be determined. Position and length of arrows and bars are approximate. See the close-up figure in Additional File 3.

The first step in the multiple-species comparisons was to depict the translocations and inversions in potato, eggplant, pepper and *Nicotiana *with respect to tomato (Figure 4 and Additional File 3). Based on the phylogenetic relationships among these species and the principle of parsimony, in most cases it was feasible to determine along which lineage a rearrangement event occurred and to deduce the putative genome arrangement of the ancestral genomes (ATPt, ATE and ATP). The genome arrangement of ATN requires a more distant species (e.g. coffee in Rubiaceae [21]) to determine and thus will not be included in the follow-up analyses. Chromosomes of the ancestral genomes were named 1-12 according to the tomato orthologs. In the following paragraphs, we will describe these rearrangements chromosome by chromosome (Figure 4 and Additional File 3).

#### T1 and its orthologs

T1 and Pt1 share the same gene order and gene content so that they both represent the ancestral arrangement in ATPt1. E1 differs from ATPt1 by one inversion, which is not seen in pepper and *Nicotiana*, therefore ATE1 has a similar arrangement to ATPt1 and the inversion is derived in E1. The pepper ortholog is P1b, part of a large chromosome P1 (P1a is orthologous to T8; also see "T8 and its orthologs"), which indicates a translocation between pepper and ATE genomes. P1b is further differentiated from ATE1 by at least two paracentric and two pericentric inversions. Nonetheless, genome arrangement of the ancestral ATP1 can only be partly determined with the more distant *Nicotiana*. On one hand, the translocation combining P1b and P1a does not exist in the *Nicotiana *genome, suggesting that translocation is specific to the pepper genome. On the other hand, the two translocations differentiating *Nicotiana *from ATP, which produced N11c, N9b and N1a, has made it difficult to identify whether ATE1 or P1b shares the same gene order with *Nicotiana *and thus the four inversions could have occurred along the lineage of either ATE or pepper.

#### T2 and its orthologs

This chromosome is the only one that has not experienced translocations along any of the five lineages. Pt2 and E2 share an inversion relative to T2, therefore Pt2 represents the ancestral arrangement in ATPt2. E2 differs from ATPt2 by two inversions, which are not seen in P2, therefore ATE2 resembles ATPt2. P2 and ATE2 are differentiated by two inversions. N2 appears to share the same gene order with T2; however, some inversions between *Nicotiana *and tomato may have not been identified because multiple sequential rearrangements after their divergence may have greatly shuffled the marker order. As a result, we cannot ascertain whether ATE2 or P2 or neither has preserved the ancestral condition. Therefore in this work we used *Nicotiana *to time the translocations but not inversions. Exceptions will be described case by case.

#### T3 and its orthologs

T3 agrees with Pt3 in both gene order and gene content and thus both have preserved the ancestral arrangement in ATPt3. E3 is differentiated from ATPt3 by at least two inversions and one translocation that inserted a T5 segment (E3b) between E3a and E3c. These arrangements are not seen in either pepper or *Nicotiana*, therefore ATE3 likely has a similar structure to ATPt3. Pepper (P4a and P3c) differs from ATE3 by two inversions and one translocation. Again, the two inversions cannot be timed with *Nicotiana *due to the reason described earlier. The *Nicotiana *orthologous counterparts are N9c and N3b--a different karyotype (or more specifically, organization of chromosome segments after translocations) from both pepper and ATE3, therefore *Nicotiana *is not helpful in timing this translocation between pepper and ATE3. Nonetheless, the breakpoint regions in pepper and *Nicotiana *overlap with each other, suggesting they may share the same breakpoint. In other words, the breakpoint may have been reused in different lineages and at different points in time.

#### T4 and its orthologs

T4 and Pt4 share the same gene order and gene content and thus both may represent the ancestral arrangement in ATPt4. E11b has three derived inversions. Pepper (P5a, P12b and P4b) likely experienced a translocation that separated P5a from the rest of the chromosome since the same translocation was not found in *Nicotiana*. P5a also differs from ATE4 by an inversion but the timing is unknown. However, distinct karyotypes among ATPt, eggplant, pepper and *Nicotiana *make it impossible to determine which lineage (ATPt, eggplant or pepper) or none of them has preserved the ancestral karyotype in ATE and ATP, although the gene order in these chromosome segments may largely be consistent with that of ATPt4 and the breakpoint may have been reused (between E11b and E4b and between P12b and P4b).

#### T5 and its orthologs

Pt5, E3b and P11a share one inversion relative to T5, so that Pt5 has preserved the arrangement in ATPt5 and the inversion is derived in T5. Eggplant experienced a translocation and multiple inversions that resulted in E3b and E10a; therefore ATE5 has a similar gene order to ATPt5. Both pepper and *Nicotiana *differ from ATE5 by one inversion, so that pepper may have preserved the ancestral gene order in the ATP genome. Eggplant (between E3b and E5a), pepper (P11a and P5b) and *Nicotiana *(N10b and N5a) possibly share the breakpoint of one translocation relative to ATPt5, but karyotype of the ATE and ATP genomes cannot be determined.

#### T6 and its orthologs

Both T6 and Pt6 have preserved the ancestral arrangement in ATPt6. E6 and P6 share one inversion relative to ATPt6 near top of the chromosome, and E6 and N6b share another inversion relative to ATPt6 near bottom of the chromosome. Therefore both ATE6 and ATP6 resemble E6 while two inversions occurred to ATPt6 and one to P6.

#### T7 and its orthologs

This chromosome is quite conserved. E7 has a derived inversion, otherwise T7, Pt7, E7 and P7 share the same gene order and gene content. Therefore, the genome arrangement in ATPt7, ATE7 and ATP7 resemble that in T7/Pt7/P7.

#### T8 and its orthologs

This chromosome is also fairly conserved. T8, Pt8 and E8 agree with each other in both gene order and gene content, therefore the genome arrangement in ATPt8 and ATE8 have been well preserved. As described in the previous section "*T1 and its orthologs*", pepper differs from the above three species by one translocation which is not seen in *Nicotiana*, therefore this translocation is likely derived in the pepper genome and ATP8 resembles ATE8.

#### T9 and its orthologs

One inversion differentiates T9 from Pt9, E9 and P9a, suggesting ATPt9 and ATE9 resemble Pt9/E9 and the gene order in ATP9 is likely the same as that in Pt9/E9. Pepper (P9a and P3a) has a translocation relative to ATE9, with the breakpoint likely shared by *Nicotiana *(between N7b and N1b), but timing of the translocation and karyotype of the ATP genome remain to be determined.

#### T10 and its orthologs

This chromosome is conserved among T10, Pt10 and P10 except for a derived inversion in T10. Therefore ATPt10, ATE10 and ATP10 have a similar arrangement to Pt10/P10. Eggplant has a quite different situation in that it experienced multiple rearrangements including one translocation and five inversions.

#### T11 and its orthologs

Pt11 is differentiated from T11 by one inversion, which is not seen in the other species, so ATPt11 resembles T11. Eggplant (E11a and E12b) and pepper (P12a and P11b) share two inversions relative to ATPt11, therefore the inversions are derived in ATPt11. Pepper has one additional inversion but its timing cannot be determined. Eggplant, pepper and *Nicotiana *share the breakpoint of a translocation relative to tomato, but again the timing of this translocation is unknown.

#### T12 and its orthologs

T12 has an inversion relative to Pt12 but shares the same gene order with the pepper orthologous counterparts, therefore T12 has preserved the genome arrangement of ATPt12. Eggplant (E10b, E12a and E5b) and pepper (P9b, P3b and P12c) each have experienced two translocations and one inversion relative to ATPt12, although these rearrangements are independent of each other. The translocation that produced E12a and the inversion on E10b are derived in eggplant, so ATE12 has a similar gene order to ATPt12. The translocation that separated P9b and P3b is derived on pepper. The inversion between P12c and T12 has an unknown timing. The other two translocations, which separated E10b and E5b as well as P3b and P12c, share a common breakpoint with *Nicotiana *(between N6b and N12b) but their timing cannot be determined.

### Rates of inversion and translocation events across the Solanaceae

**Figure 5:**
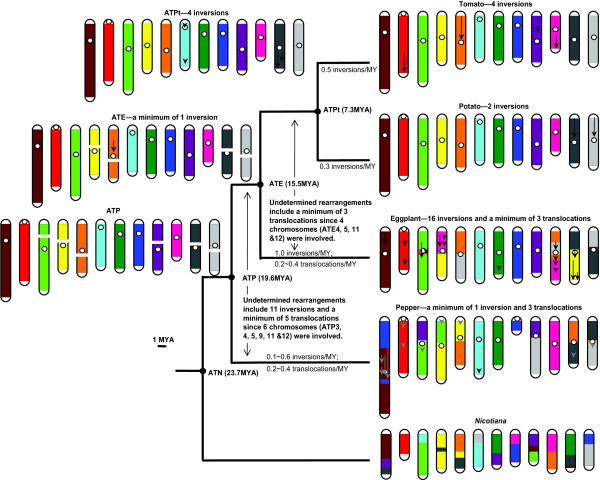
**Chromosomal evolution in the family Solanaceae**. The actual karyotype (chromosome 1-12) of tomato, potato, eggplant, pepper and *Nicotiana*, and the deduced karyotype of their MRCAs are presented. Each tomato chromosome is assigned a different color, and the orthologous counterparts in the other species are painted with the corresponding color, which therefore depicts the translocations differentiating these species. Black arrows on chromosomes represent the inversions that these species have experienced subsequent to divergence from their MRCA, i.e. inversions in tomato and potato relative to ATPt, inversions in eggplant and ATPt relative to ATE, and inversions in ATE and pepper relative to ATP. Position and length of the arrows are approximate (see Additional Files 2 for details). Grey arrows on the pepper chromosomes represent inversions between pepper and ATE but it remains to be determined along which lineage these inversions occurred. White dots indicate approximate centromere location of the tomato chromosomes [30] and putative centromere location of the eggplant and pepper chromosomes based on their synteny with tomato [9,10]. Broken chromosomes in the ATE and ATP genomes represent unknown karyotypes, in other words, organization of these chromosome segments remains to be determined.

Comparing the genetic maps of tomato, potato, eggplant, pepper and *Nicotiana *has permitted us, in many instances, to deduce the genome arrangement of their MRCAs and to determine in which lineage chromosomal rearrangements occurred. As a result, we present actual genetic maps of the extant species and deduced genetic maps of their MRCAs according to their phylogenetic relationships, and depict the possible inversion and translocation events that occurred leading to each genome (Figure 5). In summary, since these species diverged from their last MRCA, four inversions have occurred along the tomato lineage, two inversions along the potato lineage, four inversions along the ATPt lineage, 16 inversions along the eggplant lineage, and at least one inversion along each of ATE and pepper lineages as well as 11 undetermined inversions between these two lineages. The situation for translocations is more complicated. Since eggplant diverged from ATE, a T5 segment was inserted into E3, a T12 segment was inserted into E10, and markers from T10 was translocated to E4 and E10 respectively. Since pepper diverged from ATP, a non-reciprocal translocation resulted in P1 and P8, a small T4 segment was inserted into P12, and a small T12 segment was inserted into P3. In addition to these events with known timing, chromosomes 4, 5, 11 and 12 have been rearranged by translocations in the ATPt and/or eggplant genomes after they diverged from the ATE genome, and chromosomes 3, 4, 5, 9, 11 and 12 have been rearranged by translocations in the ATE and/or pepper genomes after they diverged from the ATP genome.

Using the divergence time estimated earlier, we calculated the absolute rates of inversions and translocations respectively across different lineages (Figure 5). After divergence from ATPt, the lineages leading to tomato and potato experienced 0.5 and 0.3 inversions/million years respectively. After divergence from ATE, the lineage leading to eggplant experienced 1 inversion/million years and 0.2 ~ 0.4 translocations/million years. After divergence from ATP, the lineage leading to pepper experienced 0.1 ~ 0.6 inversions/million years and 0.2 ~ 0.4 translocations/million years. These results suggested a fairly consistent rate of chromosomal rearrangements across these solanaceous species. One exception is that the eggplant lineage appears to have experienced a higher number of inversions. One possible explanation for this observation is that some inversions between ATPt and eggplant/pepper cannot be deciphered between ATPt and pepper due to larger evolutionary distance and thus the inversions were assigned into the eggplant lineage. This same factor may also lead to an underestimate of the inversion number between ATE and pepper. Nonetheless, these results provide the first general outline of the chromosomal evolution in the Solanaceae.

## Discussion

### Evaluation of the parsimony analyses

Given the phylogenetic relationships of the solanaceous species included in this study (Figure 1), we performed parsimony analyses to determine the timing of rearrangement events that differentiate these species. The results from this study can be used to highlight a number of points. First, multiple distant species are necessary for timing inversions between a pair of sister species. For example, regarding the inversion between T5 and Pt5 (Figure 4 and Additional File 3), eggplant experienced a translocation in this region which moved some markers to E10 so that it was hard to determine with either tomato or potato eggplant shared the same gene order. Nonetheless, pepper showed clear agreement with potato, thus supporting potato preserved the ancestral condition. Secondly, species with large phylogenetic distance may not be good for timing inversions. *Nicotiana *is the most distant from the other species but appears to differ from tomato by 10 inversions only, possibly because multiple sequential rearrangements shuffled the marker order to the extent that individual inversions can no longer be deciphered [11]. Among these 10 inversions, only three were shared by the other species and thus were useful for determining the time of these events. As a result, we left 11 of the inversions between ATE and pepper as events with unknown timing. Pepper, in some cases, may also be too distant to time the inversions between ATPt and eggplant, thus a higher inversion rate was estimated along the eggplant lineage, although some of the inversions may actually have occurred along the ATPt lineage or more anciently before the divergence of ATPt and eggplant. Thirdly, more species are in need to decipher the timing of translocation events. In more than half of the identified translocations (Figure 5), the species being compared each have a different karyotype (or organization of chromosome segments after translocations) and thus we could not assign these events into a specific lineage or deduce the ancestral chromosome arrangement. One possible solution is to construct genetic maps and comparative maps of more solanaceous species within the phylogenetic distance between tomato and *Nicotiana*.

### Possible reuse of chromosomal breakpoints for independent translocation events

Among all the identified translocations differentiating the solanaceous species studied, we found that some events have overlapping breakpoint regions, e.g. the two independent translocations relative to T3, which produced P4a and P3c for pepper and N9c and N3b for *Nicotiana *respectively (Figure 4 and Additional File 3). However, since resolution of the breakpoint regions is limited by the density of synteny markers, we cannot ascertain whether the breakpoints are in the exact same position. Similar cases were found for the translocation events relative to T3, T4, T5, T9, T11 and T12 (Figure 4 and Additional File 3). Such phenomenon--reuse of breakpoints, has been reported as a common phenomenon in mammals [22-25]. Pevzner and Tesler [25] described mammalian genomes as mosaics of rearrangement hotspots and conserved segments, and proposed a fragile breakage model which was opposed to the widely accepted random breakage theory by Nadeau and Taylor [26]. It was reported that ~20% of breakpoint regions were reused during mammalian evolution and the sites were enriched for centromeres [24]. Interestingly and consistently, five out of the six cases in our finding (except for the one on T3) also had breakpoints near the predicted position of the tomato centromere (Figure 4 and Additional File 3).

### Comparing rates of chromosomal evolution between Solanaceae and other plant and animal taxa

Comparing the maps of tomato, potato, eggplant and pepper genomes provided an opportunity to estimate rates of chromosomal evolution in the Solanaceae, resulting in an estimate of 0.1 ~ 1 inversions per million years and 0.2 ~ 0.4 translocations per million years across different species, in other words, 0.03 ~ 0.12 rearrangements per chromosome per million years. It was hard to make precise comparisons of this rate and those from other families due to differences in mapping techniques, map resolutions, criteria to identify rearrangements and methods of estimating divergence time. Nonetheless, this rate in the Solanaceae is comparable with that in the plant families Poaceae, Malvaceae and Brassicaceae as well as many mammals [24,27,28], although the rate is lower than that reported for some fast evolving taxa such as the genus *Arabidopsis *[27,29]. Given the largely constant chromosome number in the Solanaceae and similar rates of chromosomal evolution across the solanaceous species studied in this work, we consider that the Solanaceae has a modest rate of chromosomal evolution and the rate is likely shared by many solanaceous species.

## Conclusions

Comparative genome studies have been performed widely for solanaceous crops, mostly between pairs of species. Recently, a large set of single-copy conserved orthologous (COSII) markers has provided an opportunity to combine data from multiple species and to describe the overall patterns of chromosomal evolution for the whole family. In the research described herein, we have taken advantage of COSII studies in tomato, potato, eggplant, pepper and diploid *Nicotiana *species, to deduce the broad features and outcomes of chromosomal evolution in the Solanaceae over the past 30 million years. The results reveal a modest and consistent rate (0.03 ~ 0.12 rearrangements per chromosome per million years) of chromosomal changes across the family, with a higher frequency of inversions than translocations. We have also identified hot spots of chromosomal breakages, supporting the notion that chromosomal rearrangement breakpoints are not randomly distributed. Finally we have reconstructed the most likely genome configuration for the ancestors of these solanaceous species. This study thus provides the first broad overview of chromosomal evolution in the family Solanaceae, and one of the most detailed thus far for plant families.

## Authors' contributions

SDT and FW conceived of the study. FW carried out the data analysis. FW and SDT drafted the manuscript. Both authors read and approved the final manuscript.

## Supplementary Material

Additional file 1 **Figure S1 - Molecular dating of divergence time for several solanaceous species**. A previously published dataset [3] was used to reconstruct the maximum-likelihood tree, and the divergence time was estimated using the non-parametric rate smoothing method (see Methods). The calibration point was 86MYA for tomato-coffee split [15]. Click here for file

Additional file 2 **Figure S2 - Chromosomal rearrangements in the genomes of eggplant, pepper and *Nicotiana *with respect to the tomato genome**. Genetic maps are modified from published work [9-11] with kind permission from Springer Science+Business Media. E1-12 represents the 12 eggplant chromosomes, P1-12 for pepper, and N1-12 for the MRCA of *N. tomentosiformis *and *N. acuminata*. Each tomato chromosome is assigned a different color (see color codes in the figure) and the orthologous chromosome segment(s) in eggplant, pepper and *Nicotiana *are painted with the same color. Putative centromere positions of eggplant and pepper chromosomes are based on their synteny with tomato and indicated by a white dot. It was not possible to determine the centromere positions for the *Nicotiana *chromosomes due to complex syntenic relationships with tomato. An arrow beside a chromosome indicates an inversion relative to tomato. A black bar indicates the breakpoint region of a translocation, and the chromosome is divided into segments (a-c) accordingly to facilitate comparisons of multiple species. The marker pair used to define the breakpoint region (i.e. the two adjacent markers mapped to different tomato chromosomes) or to define the borders of an inversion is highlighted in red. The map of the MRCA of *N. tomentosiformis *and *N. acuminata *is deduced based on the comparative maps of *N. tomentosiformis, N. acuminata *and tomato, and is not presented directly. The actual *N. tomentosiformis *map is presented, and the four inversions relative to its MRCA are indicated by dash arrows and the border markers are underlined. Click here for file

Additional file 3 **Figure S3 - Comparative maps of several solanaceous species and the deduced genome arrangement of MRCAs (close-up of Figure **4**)**. Designation of chromosome and chromosome segment (a-c) as well as color codes follow Additional File 2. Nomenclature of MRCAs (ATPt, ATE and ATP) follows Figure 1. Maps of non-tomato species are depicted in a comparative way to the tomato map as follows. A black arrow depicts an inversion relative to tomato (a grey arrow for an uncertain inversion). A black bar depicts the breakpoint region of a translocation relative to tomato (a grey bar for an uncertain translocation). Two black bars connected by a curve indicate that the segment in between is excised in a translocation while the remained parts stay together, e.g. E10a is embedded in E3b. "1+" (or "2+") on a single arrow indicates that the region has experienced at least one (or two) inversions but the exact number remains to be determined. Markers displayed on the tomato map were used to define breakpoint regions of translocations and borders of inversions. The prefix (in parentheses) of a marker name specifies at which non-tomato maps the marker locates (E = eggplant, P = pepper, N = *Nicotiana*). Due to a different tomato map used for tomato-potato comparison, location and length of inversions on the potato map are approximate [3,8]. White dots indicate the approximate centromere location of the tomato chromosomes. Click here for file
