# Supplementary material for: Chromosomal evolution in the plant family Solanaceae
Source: BMC Genomics. 2010 Mar 17;11:182. doi: 10.1186/1471-2164-11-182 (PMC2847972; doi:10.1186/1471-2164-11-182)
Supplement: Additional file 1 — Figure S1 - Molecular dating of divergence time for several solanaceous species. A previously published dataset [3] was used to reconstruct the maximum-likelihood tree, and the divergence time was estimated using the non-parametric rate smoothing method (see Methods). The calibration point was 86MYA for tomato-coffee split [15]. [file 1471-2164-11-182-S1.PPT]

## Slide 1
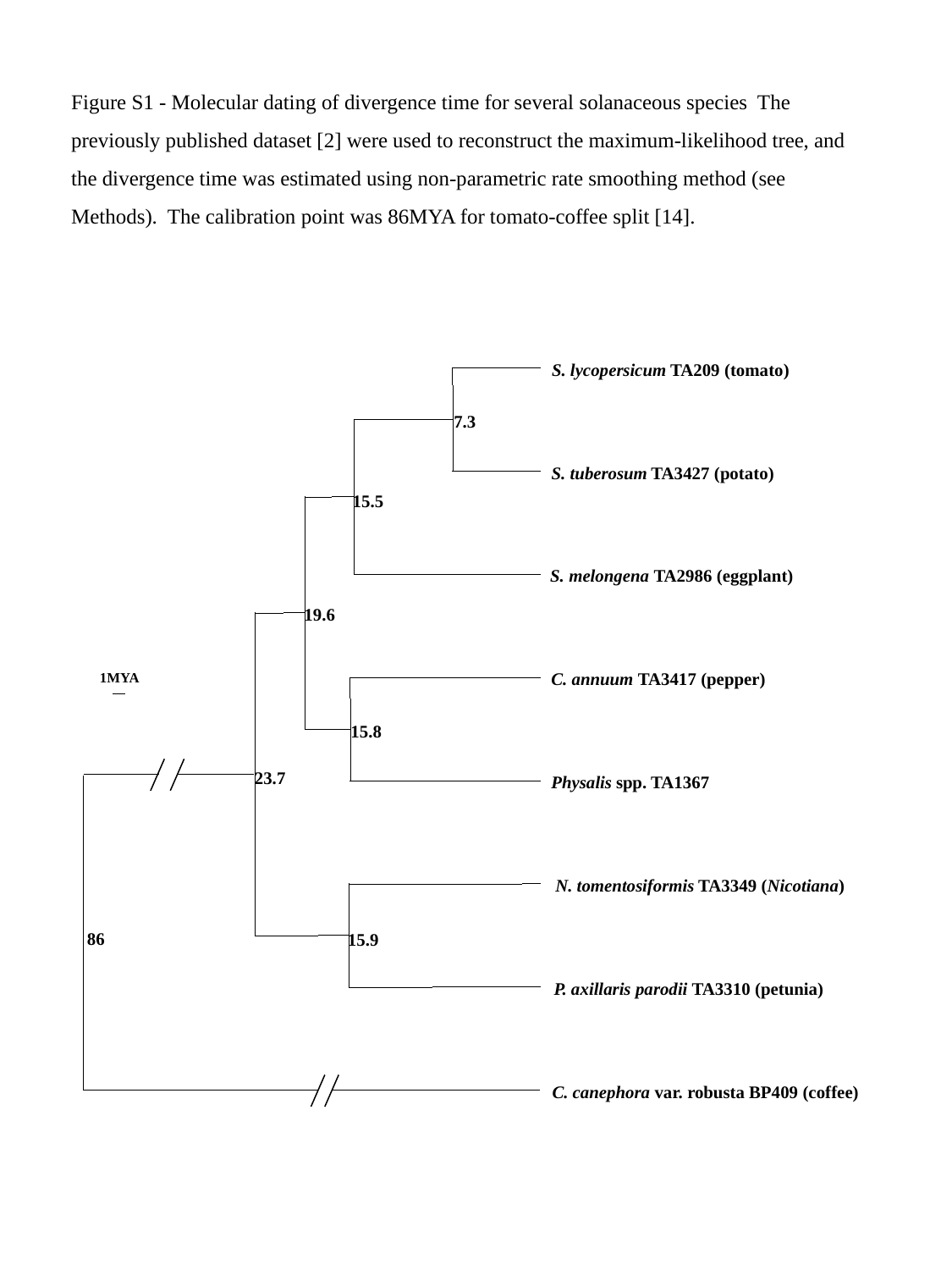

Figure S1 - Molecular dating of divergence time for several solanaceous species The previously published dataset [2] were used to reconstruct the maximum-likelihood tree, and the divergence time was estimated using non-parametric rate smoothing method (see Methods). The calibration point was 86MYA for tomato-coffee split [14].
S. lycopersicum TA209 (tomato)
S. tuberosum TA3427 (potato)
S. melongena TA2986 (eggplant)
C. annuum TA3417 (pepper)
Physalis spp. TA1367
N. tomentosiformis TA3349 (Nicotiana)
P. axillaris parodii TA3310 (petunia)
C. canephora var. robusta BP409 (coffee)
7.3
15.5
19.6
1MYA
15.8
23.7
86
15.9
