# Supplementary material for: Chromosomal evolution in the plant family Solanaceae
Source: BMC Genomics. 2010 Mar 17;11:182. doi: 10.1186/1471-2164-11-182 (PMC2847972; doi:10.1186/1471-2164-11-182)
Supplement: Additional file 2 — Figure S2 - Chromosomal rearrangements in the genomes of eggplant, pepper and Nicotiana with respect to the tomato genome. Genetic maps are modified from published work [9-11] with kind permission from Springer Science+Business Media. E1-12 represents the 12 eggplant chromosomes, P1-12 for pepper, and N1-12 for the MRCA of N. tomentosiformis and N. acuminata. Each tomato chromosome is assigned a different color (see color codes in the figure) and the orthologous chromosome segment(s) in eggplant, pepper and Nicotiana are painted with the same color. Putative centromere positions of eggplant and pepper chromosomes are based on their synteny with tomato and indicated by a white dot. It was not possible to determine the centromere positions for the Nicotiana chromosomes due to complex syntenic relationships with tomato. An arrow beside a chromosome indicates an inversion relative to tomato. A black bar indicates the breakpoint region of a translocation, and the chromosome is divided into segments (a-c) accordingly to facilitate comparisons of multiple species. The marker pair used to define the breakpoint region (i.e. the two adjacent markers mapped to different tomato chromosomes) or to define the borders of an inversion is highlighted in red. The map of the MRCA of N. tomentosiformis and N. acuminata is deduced based on the comparative maps of N. tomentosiformis, N. acuminata and tomato, and is not presented directly. The actual N. tomentosiformis map is presented, and the four inversions relative to its MRCA are indicated by dash arrows and the border markers are underlined. [file 1471-2164-11-182-S2.PPT]

## Slide 1
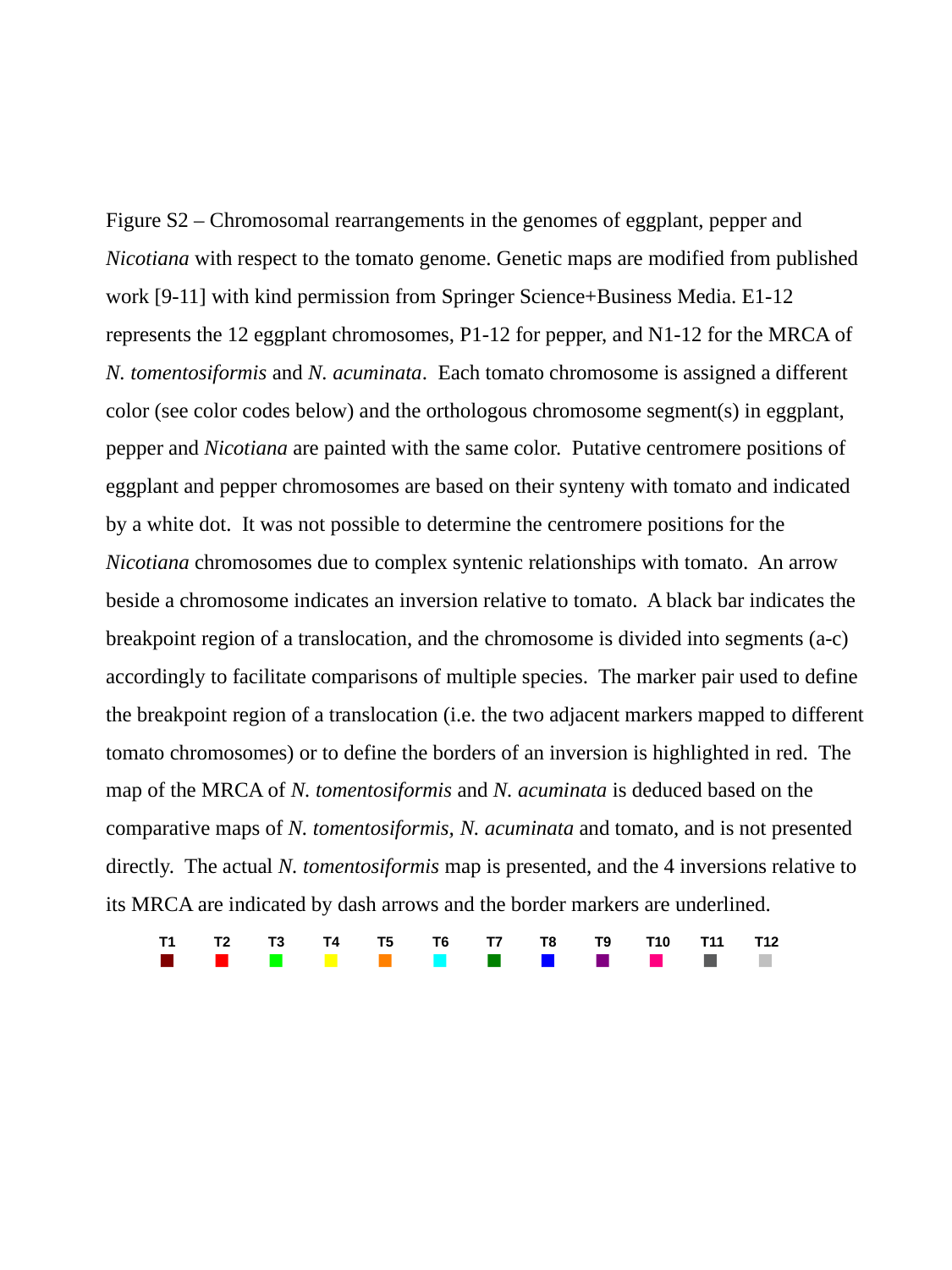

Figure S2 – Chromosomal rearrangements in the genomes of eggplant, pepper and Nicotiana with respect to the tomato genome. Genetic maps are modified from published work [9-11] with kind permission from Springer Science+Business Media. E1-12 represents the 12 eggplant chromosomes, P1-12 for pepper, and N1-12 for the MRCA of N. tomentosiformis and N. acuminata. Each tomato chromosome is assigned a different color (see color codes below) and the orthologous chromosome segment(s) in eggplant, pepper and Nicotiana are painted with the same color. Putative centromere positions of eggplant and pepper chromosomes are based on their synteny with tomato and indicated by a white dot. It was not possible to determine the centromere positions for the Nicotiana chromosomes due to complex syntenic relationships with tomato. An arrow beside a chromosome indicates an inversion relative to tomato. A black bar indicates the breakpoint region of a translocation, and the chromosome is divided into segments (a-c) accordingly to facilitate comparisons of multiple species. The marker pair used to define the breakpoint region of a translocation (i.e. the two adjacent markers mapped to different tomato chromosomes) or to define the borders of an inversion is highlighted in red. The map of the MRCA of N. tomentosiformis and N. acuminata is deduced based on the comparative maps of N. tomentosiformis, N. acuminata and tomato, and is not presented directly. The actual N. tomentosiformis map is presented, and the 4 inversions relative to its MRCA are indicated by dash arrows and the border markers are underlined.
T1
T2
T3
T4
T5
T6
T7
T8
T9
T10
T11
T12

## Slide 2
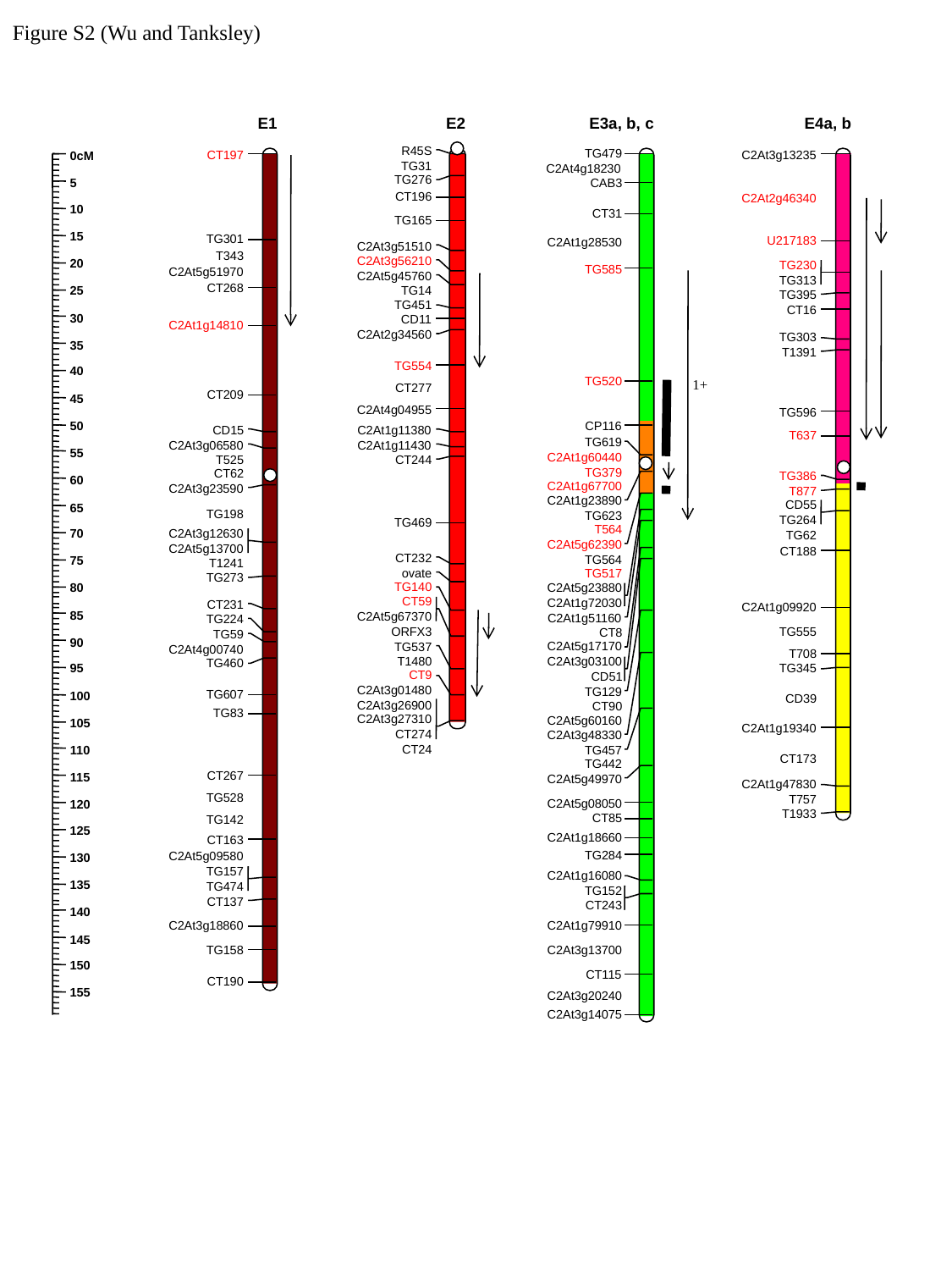

Figure S2 (Wu and Tanksley)
E1
CT197
TG301
T343
C2At5g51970
CT268
C2At1g14810
CT209
CD15
C2At3g06580
T525
CT62
C2At3g23590
TG198
C2At3g12630
C2At5g13700
T1241
TG273
CT231
TG224
TG59
C2At4g00740
TG460
TG607
TG83
CT267
TG528
TG142
CT163
C2At5g09580
TG157
TG474
CT137
C2At3g18860
TG158
CT190
E2
R45S
TG31
TG276
CT196
TG165
C2At3g51510
C2At3g56210
C2At5g45760
TG14
TG451
CD11
C2At2g34560
TG554
CT277
C2At4g04955
C2At1g11380
C2At1g11430
CT244
TG469
CT232
ovate
TG140
CT59
C2At5g67370
ORFX3
TG537
T1480
CT9
C2At3g01480
C2At3g26900
C2At3g27310
CT274
CT24
E3a, b, c
TG479
C2At4g18230
CAB3
CT31
C2At1g28530
TG585
1+
TG520
CP116
TG619
C2At1g60440
TG379
C2At1g67700
C2At1g23890
TG623
T564
C2At5g62390
TG564
TG517
C2At5g23880
C2At1g72030
C2At1g51160
CT8
C2At5g17170
C2At3g03100
CD51
TG129
CT90
C2At5g60160
C2At3g48330
TG457
TG442
C2At5g49970
C2At5g08050
CT85
C2At1g18660
TG284
C2At1g16080
TG152
CT243
C2At1g79910
C2At3g13700
CT115
C2At3g20240
C2At3g14075
E4a, b
C2At3g13235
C2At2g46340
U217183
TG230
TG313
TG395
CT16
TG303
T1391
TG596
T637
TG386
T877
CD55
TG264
TG62
CT188
C2At1g09920
TG555
T708
TG345
CD39
C2At1g19340
CT173
C2At1g47830
T757
T1933
0cM
5
10
15
20
25
30
35
40
45
50
55
60
65
70
75
80
85
90
95
100
105
110
115
120
125
130
135
140
145
150
155

## Slide 3
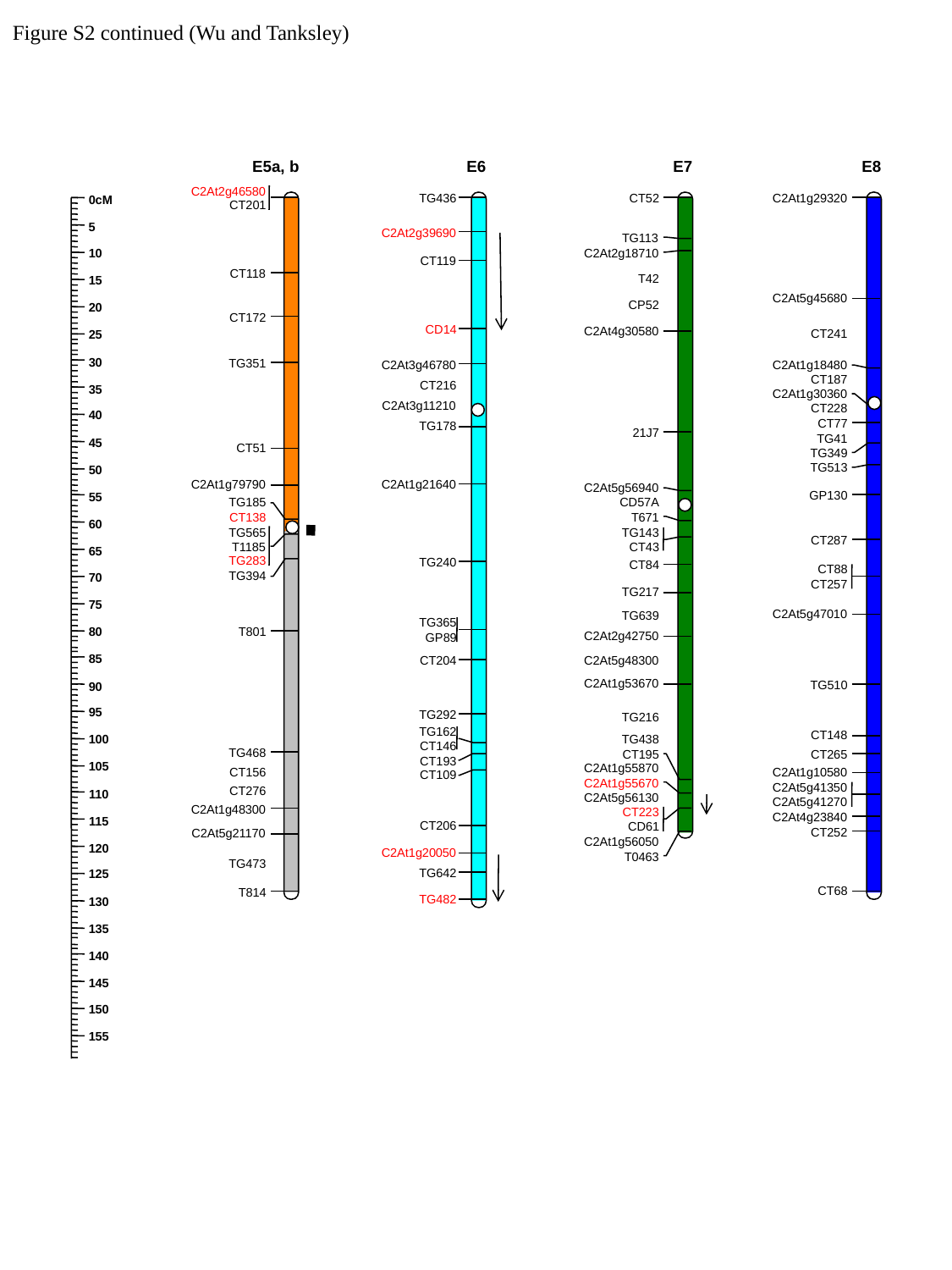

Figure S2 continued (Wu and Tanksley)
E5a, b
C2At2g46580
CT201
CT118
CT172
TG351
CT51
C2At1g79790
TG185
CT138
TG565
T1185
TG283
TG394
T801
TG468
CT156
CT276
C2At1g48300
C2At5g21170
TG473
T814
E6
TG436
C2At2g39690
CT119
CD14
C2At3g46780
CT216
C2At3g11210
TG178
C2At1g21640
TG240
TG365
GP89
CT204
TG292
TG162
CT146
CT193
CT109
CT206
C2At1g20050
TG642
TG482
E7
CT52
TG113
C2At2g18710
T42
CP52
C2At4g30580
21J7
C2At5g56940
CD57A
T671
TG143
CT43
CT84
TG217
TG639
C2At2g42750
C2At5g48300
C2At1g53670
TG216
TG438
CT195
C2At1g55870
C2At1g55670
C2At5g56130
CT223
CD61
C2At1g56050
T0463
E8
C2At1g29320
C2At5g45680
CT241
C2At1g18480
CT187
C2At1g30360
CT228
CT77
TG41
TG349
TG513
GP130
CT287
CT88
CT257
C2At5g47010
TG510
CT148
CT265
C2At1g10580
C2At5g41350
C2At5g41270
C2At4g23840
CT252
CT68
0cM
5
10
15
20
25
30
35
40
45
50
55
60
65
70
75
80
85
90
95
100
105
110
115
120
125
130
135
140
145
150
155

## Slide 4
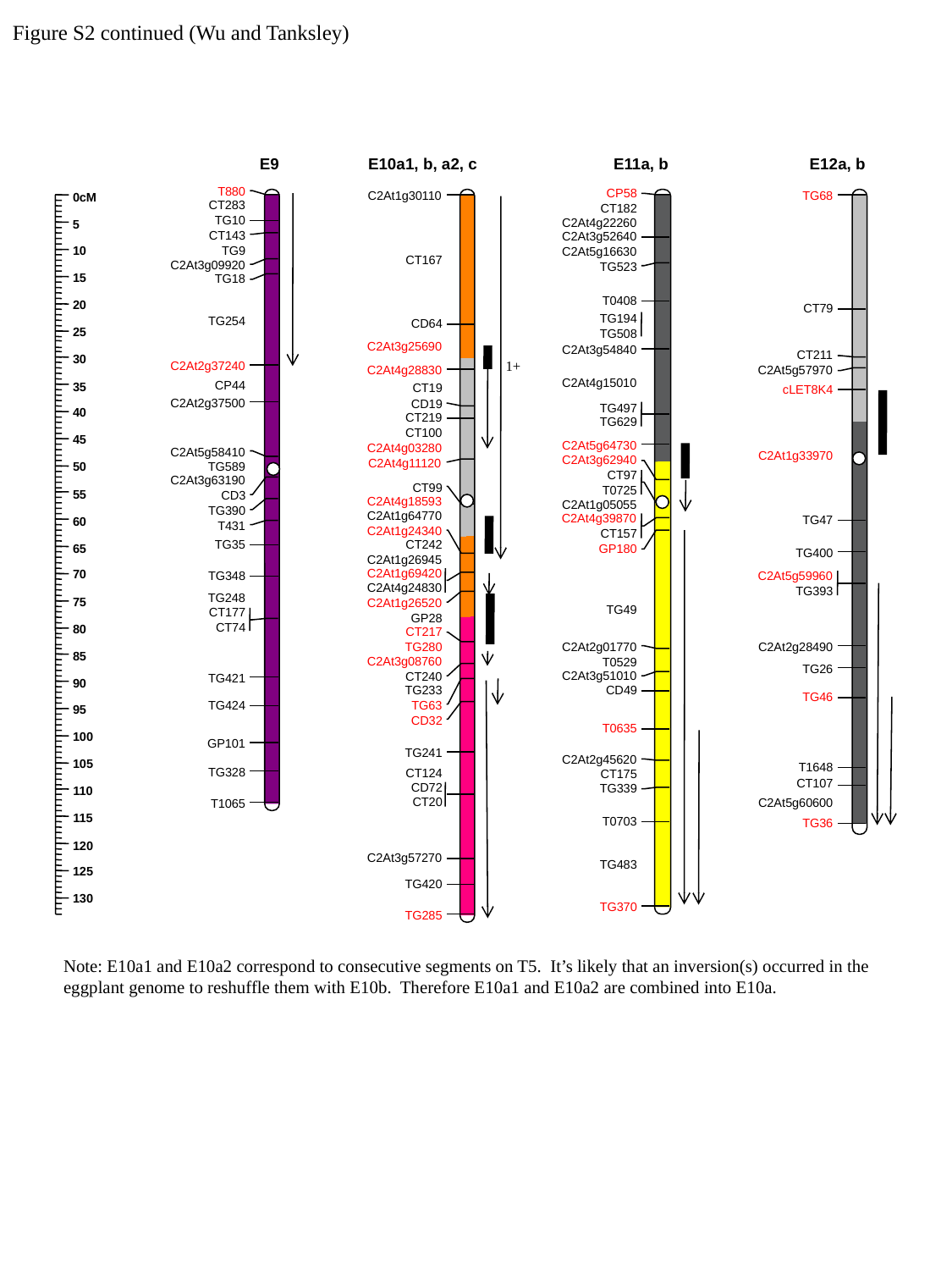

Figure S2 continued (Wu and Tanksley)
E9
T880
CT283
TG10
CT143
TG9
C2At3g09920
TG18
TG254
C2At2g37240
CP44
C2At2g37500
C2At5g58410
TG589
C2At3g63190
CD3
TG390
T431
TG35
TG348
TG248
CT177
CT74
TG421
TG424
GP101
TG328
T1065
E10a1, b, a2, c
C2At1g30110
CT167
CD64
C2At3g25690
C2At4g28830
CT19
CD19
CT219
CT100
C2At4g03280
C2At4g11120
CT99
C2At4g18593
C2At1g64770
C2At1g24340
CT242
C2At1g26945
C2At1g69420
C2At4g24830
C2At1g26520
GP28
CT217
TG280
C2At3g08760
CT240
TG233
TG63
CD32
TG241
CT124
CD72
CT20
C2At3g57270
TG420
TG285
E11a, b
CP58
CT182
C2At4g22260
C2At3g52640
C2At5g16630
TG523
T0408
TG194
TG508
C2At3g54840
C2At4g15010
TG497
TG629
C2At5g64730
C2At3g62940
CT97
T0725
C2At1g05055
C2At4g39870
CT157
GP180
TG49
C2At2g01770
T0529
C2At3g51010
CD49
T0635
C2At2g45620
CT175
TG339
T0703
TG483
TG370
E12a, b
TG68
CT79
CT211
C2At5g57970
cLET8K4
C2At1g33970
TG47
TG400
C2At5g59960
TG393
C2At2g28490
TG26
TG46
T1648
CT107
C2At5g60600
TG36
0cM
5
10
15
20
25
30
35
40
45
50
55
60
65
70
75
80
85
90
95
100
105
110
115
120
125
130
1+
Note: E10a1 and E10a2 correspond to consecutive segments on T5. It’s likely that an inversion(s) occurred in the eggplant genome to reshuffle them with E10b. Therefore E10a1 and E10a2 are combined into E10a.

## Slide 5
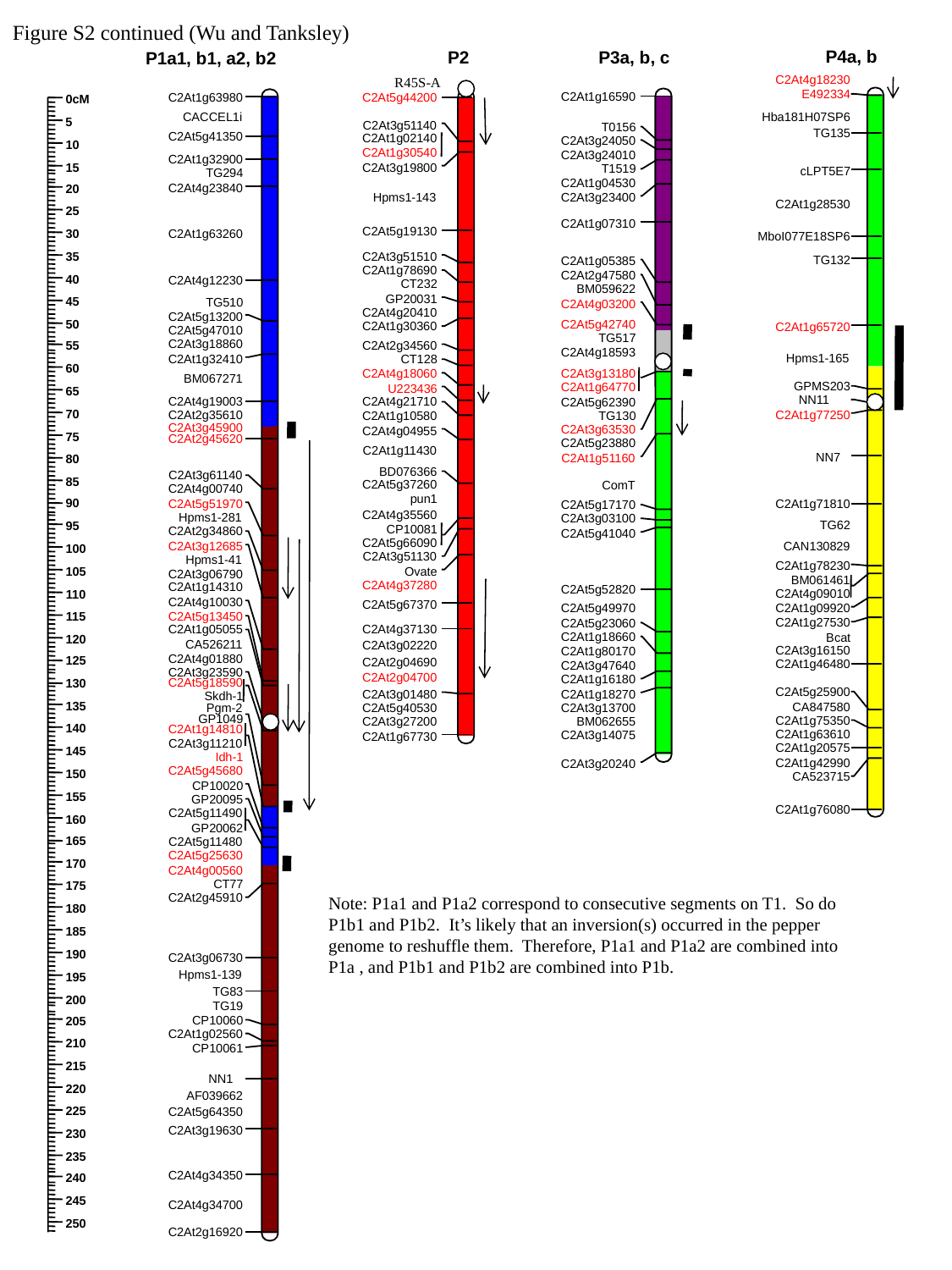

Figure S2 continued (Wu and Tanksley)
P4a, b
C2At4g18230
E492334
Hba181H07SP6
TG135
cLPT5E7
C2At1g28530
MboI077E18SP6
TG132
C2At1g65720
Hpms1-165
GPMS203
NN11
C2At1g77250
NN7
C2At1g71810
TG62
CAN130829
C2At1g78230
BM061461
C2At4g09010
C2At1g09920
C2At1g27530
Bcat
C2At3g16150
C2At1g46480
C2At5g25900
CA847580
C2At1g75350
C2At1g63610
C2At1g20575
C2At1g42990
CA523715
C2At1g76080
P2
R45S-A
C2At5g44200
C2At3g51140
C2At1g02140
C2At1g30540
C2At3g19800
Hpms1-143
C2At5g19130
C2At3g51510
C2At1g78690
CT232
GP20031
C2At4g20410
C2At1g30360
C2At2g34560
CT128
C2At4g18060
U223436
C2At4g21710
C2At1g10580
C2At4g04955
C2At1g11430
BD076366
C2At5g37260
pun1
C2At4g35560
CP10081
C2At5g66090
C2At3g51130
Ovate
C2At4g37280
C2At5g67370
C2At4g37130
C2At3g02220
C2At2g04690
C2At2g04700
C2At3g01480
C2At5g40530
C2At3g27200
C2At1g67730
P3a, b, c
C2At1g16590
T0156
C2At3g24050
C2At3g24010
T1519
C2At1g04530
C2At3g23400
C2At1g07310
C2At1g05385
C2At2g47580
BM059622
C2At4g03200
C2At5g42740
TG517
C2At4g18593
C2At3g13180
C2At1g64770
C2At5g62390
TG130
C2At3g63530
C2At5g23880
C2At1g51160
ComT
C2At5g17170
C2At3g03100
C2At5g41040
C2At5g52820
C2At5g49970
C2At5g23060
C2At1g18660
C2At1g80170
C2At3g47640
C2At1g16180
C2At1g18270
C2At3g13700
BM062655
C2At3g14075
C2At3g20240
P1a1, b1, a2, b2
C2At1g63980
CACCEL1i
C2At5g41350
C2At1g32900
TG294
C2At4g23840
C2At1g63260
C2At4g12230
TG510
C2At5g13200
C2At5g47010
C2At3g18860
C2At1g32410
BM067271
C2At4g19003
C2At2g35610
C2At3g45900
C2At2g45620
C2At3g61140
C2At4g00740
C2At5g51970
Hpms1-281
C2At2g34860
C2At3g12685
Hpms1-41
C2At3g06790
C2At1g14310
C2At4g10030
C2At5g13450
C2At1g05055
CA526211
C2At4g01880
C2At3g23590
C2At5g18590
Skdh-1
Pgm-2
GP1049
C2At1g14810
C2At3g11210
Idh-1
C2At5g45680
CP10020
GP20095
C2At5g11490
GP20062
C2At5g11480
C2At5g25630
C2At4g00560
CT77
C2At2g45910
C2At3g06730
Hpms1-139
TG83
TG19
CP10060
C2At1g02560
CP10061
NN1
AF039662
C2At5g64350
C2At3g19630
C2At4g34350
C2At4g34700
C2At2g16920
0cM
5
10
15
20
25
30
35
40
45
50
55
60
65
70
75
80
85
90
95
100
105
110
115
120
125
130
135
140
145
150
155
160
165
170
175
180
185
190
195
200
205
210
215
220
225
230
235
240
245
250
Note: P1a1 and P1a2 correspond to consecutive segments on T1. So do P1b1 and P1b2. It’s likely that an inversion(s) occurred in the pepper genome to reshuffle them. Therefore, P1a1 and P1a2 are combined into P1a , and P1b1 and P1b2 are combined into P1b.

## Slide 6
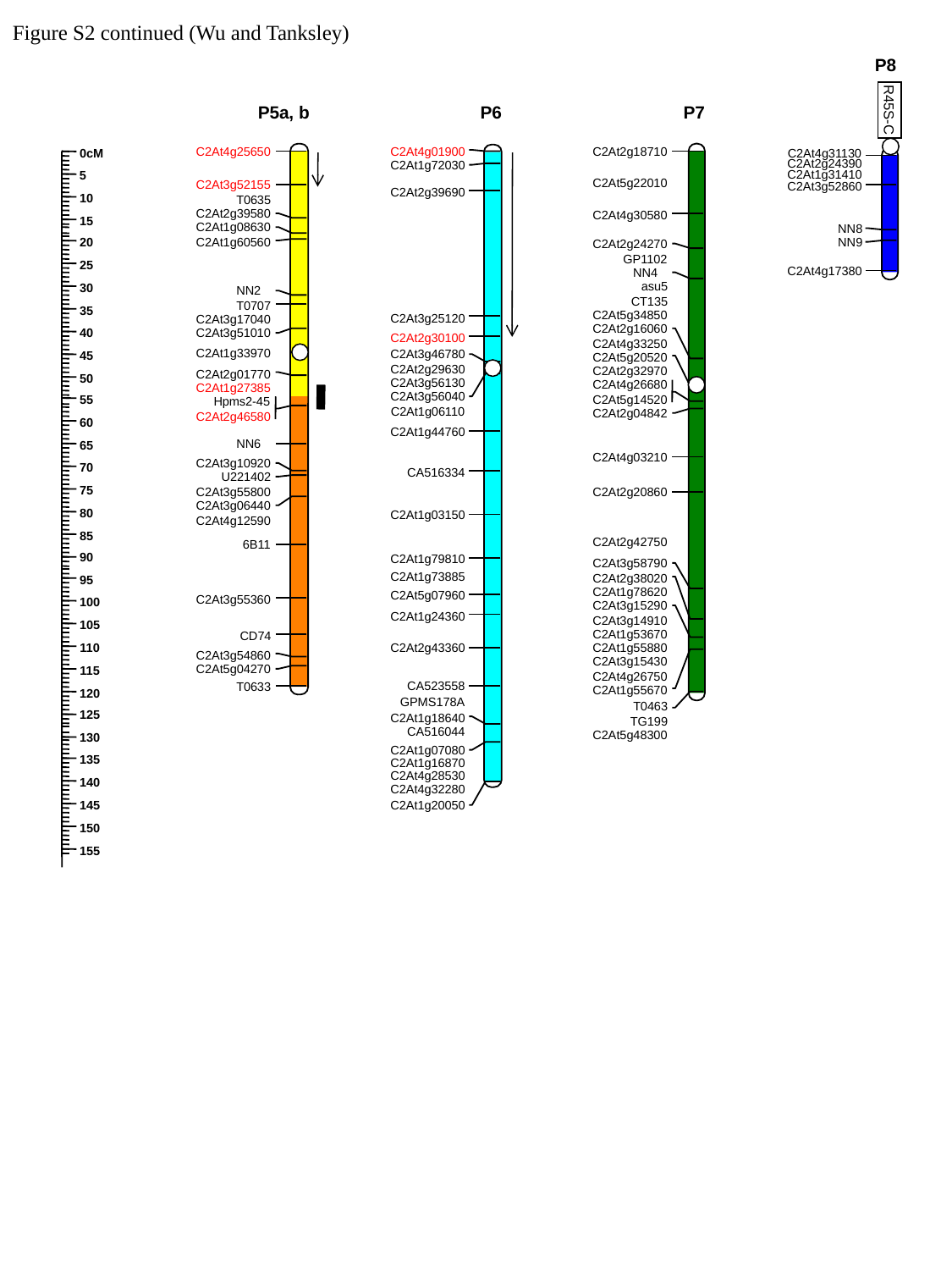

Figure S2 continued (Wu and Tanksley)
P8
R45S-C
C2At4g31130
C2At2g24390
C2At1g31410
C2At3g52860
NN8
NN9
C2At4g17380
P5a, b
C2At4g25650
C2At3g52155
T0635
C2At2g39580
C2At1g08630
C2At1g60560
NN2
T0707
C2At3g17040
C2At3g51010
C2At1g33970
C2At2g01770
C2At1g27385
Hpms2-45
C2At2g46580
NN6
C2At3g10920
U221402
C2At3g55800
C2At3g06440
C2At4g12590
6B11
C2At3g55360
CD74
C2At3g54860
C2At5g04270
T0633
P6
C2At4g01900
C2At1g72030
C2At2g39690
C2At3g25120
C2At2g30100
C2At3g46780
C2At2g29630
C2At3g56130
C2At3g56040
C2At1g06110
C2At1g44760
CA516334
C2At1g03150
C2At1g79810
C2At1g73885
C2At5g07960
C2At1g24360
C2At2g43360
CA523558
GPMS178A
C2At1g18640
CA516044
C2At1g07080
C2At1g16870
C2At4g28530
C2At4g32280
C2At1g20050
P7
C2At2g18710
C2At5g22010
C2At4g30580
C2At2g24270
GP1102
NN4
asu5
CT135
C2At5g34850
C2At2g16060
C2At4g33250
C2At5g20520
C2At2g32970
C2At4g26680
C2At5g14520
C2At2g04842
C2At4g03210
C2At2g20860
C2At2g42750
C2At3g58790
C2At2g38020
C2At1g78620
C2At3g15290
C2At3g14910
C2At1g53670
C2At1g55880
C2At3g15430
C2At4g26750
C2At1g55670
T0463
TG199
C2At5g48300
0cM
5
10
15
20
25
30
35
40
45
50
55
60
65
70
75
80
85
90
95
100
105
110
115
120
125
130
135
140
145
150
155

## Slide 7
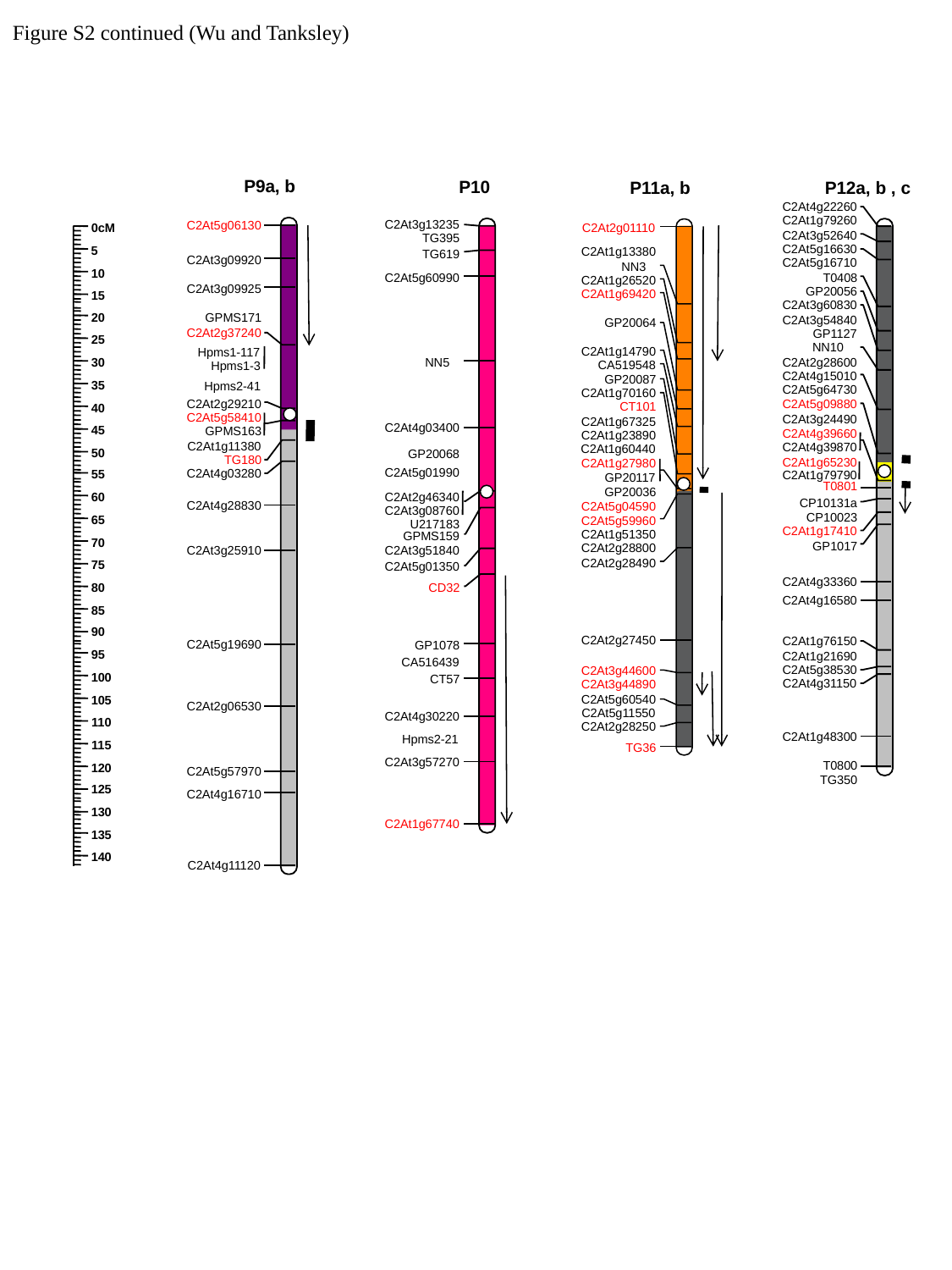

Figure S2 continued (Wu and Tanksley)
P9a, b
C2At5g06130
C2At3g09920
C2At3g09925
GPMS171
C2At2g37240
Hpms1-117
Hpms1-3
Hpms2-41
C2At2g29210
C2At5g58410
GPMS163
C2At1g11380
TG180
C2At4g03280
C2At4g28830
C2At3g25910
C2At5g19690
C2At2g06530
C2At5g57970
C2At4g16710
C2At4g11120
P10
C2At3g13235
TG395
TG619
C2At5g60990
NN5
C2At4g03400
GP20068
C2At5g01990
C2At2g46340
C2At3g08760
U217183
GPMS159
C2At3g51840
C2At5g01350
CD32
GP1078
CA516439
CT57
C2At4g30220
Hpms2-21
C2At3g57270
C2At1g67740
P11a, b
C2At2g01110
C2At1g13380
NN3
C2At1g26520
C2At1g69420
GP20064
C2At1g14790
CA519548
GP20087
C2At1g70160
CT101
C2At1g67325
C2At1g23890
C2At1g60440
C2At1g27980
GP20117
GP20036
C2At5g04590
C2At5g59960
C2At1g51350
C2At2g28800
C2At2g28490
C2At2g27450
C2At3g44600
C2At3g44890
C2At5g60540
C2At5g11550
C2At2g28250
TG36
P12a, b , c
C2At4g22260
C2At1g79260
C2At3g52640
C2At5g16630
C2At5g16710
T0408
GP20056
C2At3g60830
C2At3g54840
GP1127
NN10
C2At2g28600
C2At4g15010
C2At5g64730
C2At5g09880
C2At3g24490
C2At4g39660
C2At4g39870
C2At1g65230
C2At1g79790
T0801
CP10131a
CP10023
C2At1g17410
GP1017
C2At4g33360
C2At4g16580
C2At1g76150
C2At1g21690
C2At5g38530
C2At4g31150
C2At1g48300
T0800
TG350
0cM
5
10
15
20
25
30
35
40
45
50
55
60
65
70
75
80
85
90
95
100
105
110
115
120
125
130
135
140

## Slide 8
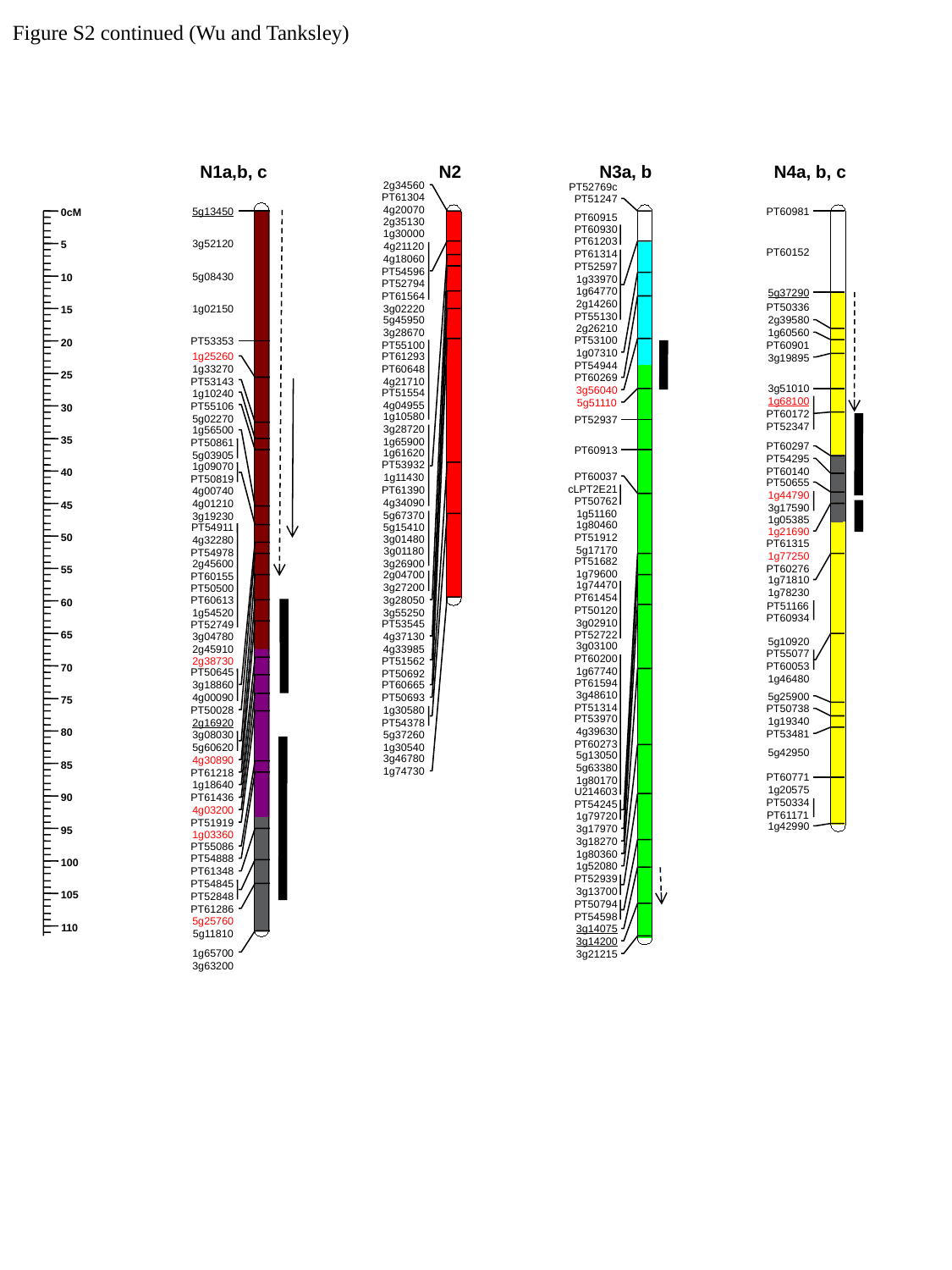

Figure S2 continued (Wu and Tanksley)
N1a,b, c
5g13450
3g52120
5g08430
1g02150
PT53353
1g25260
1g33270
PT53143
1g10240
PT55106
5g02270
1g56500
PT50861
5g03905
1g09070
PT50819
4g00740
4g01210
3g19230
PT54911
4g32280
PT54978
2g45600
PT60155
PT50500
PT60613
1g54520
PT52749
3g04780
2g45910
2g38730
PT50645
3g18860
4g00090
PT50028
2g16920
3g08030
5g60620
4g30890
PT61218
1g18640
PT61436
4g03200
PT51919
1g03360
PT55086
PT54888
PT61348
PT54845
PT52848
PT61286
5g25760
5g11810
1g65700
3g63200
N2
2g34560
PT61304
4g20070
2g35130
1g30000
4g21120
4g18060
PT54596
PT52794
PT61564
3g02220
5g45950
3g28670
PT55100
PT61293
PT60648
4g21710
PT51554
4g04955
1g10580
3g28720
1g65900
1g61620
PT53932
1g11430
PT61390
4g34090
5g67370
5g15410
3g01480
3g01180
3g26900
2g04700
3g27200
3g28050
3g55250
PT53545
4g37130
4g33985
PT51562
PT50692
PT60665
PT50693
1g30580
PT54378
5g37260
1g30540
3g46780
1g74730
N3a, b
N4a, b, c
PT60981
PT60152
5g37290
PT50336
2g39580
1g60560
PT60901
3g19895
3g51010
1g68100
PT60172
PT52347
PT60297
PT54295
PT60140
PT50655
1g44790
3g17590
1g05385
1g21690
PT61315
1g77250
PT60276
1g71810
1g78230
PT51166
PT60934
5g10920
PT55077
PT60053
1g46480
5g25900
PT50738
1g19340
PT53481
5g42950
PT60771
1g20575
PT50334
PT61171
1g42990
PT52769c
PT51247
0cM
5
10
15
20
25
30
35
40
45
50
55
60
65
70
75
80
85
90
95
100
105
110
PT60915
PT60930
PT61203
PT61314
PT52597
1g33970
1g64770
2g14260
PT55130
2g26210
PT53100
1g07310
PT54944
PT60269
3g56040
5g51110
PT52937
PT60913
PT60037
cLPT2E21
PT50762
1g51160
1g80460
PT51912
5g17170
PT51682
1g79600
1g74470
PT61454
PT50120
3g02910
PT52722
3g03100
PT60200
1g67740
PT61594
3g48610
PT51314
PT53970
4g39630
PT60273
5g13050
5g63380
1g80170
U214603
PT54245
1g79720
3g17970
3g18270
1g80360
1g52080
PT52939
3g13700
PT50794
PT54598
3g14075
3g14200
3g21215

## Slide 9
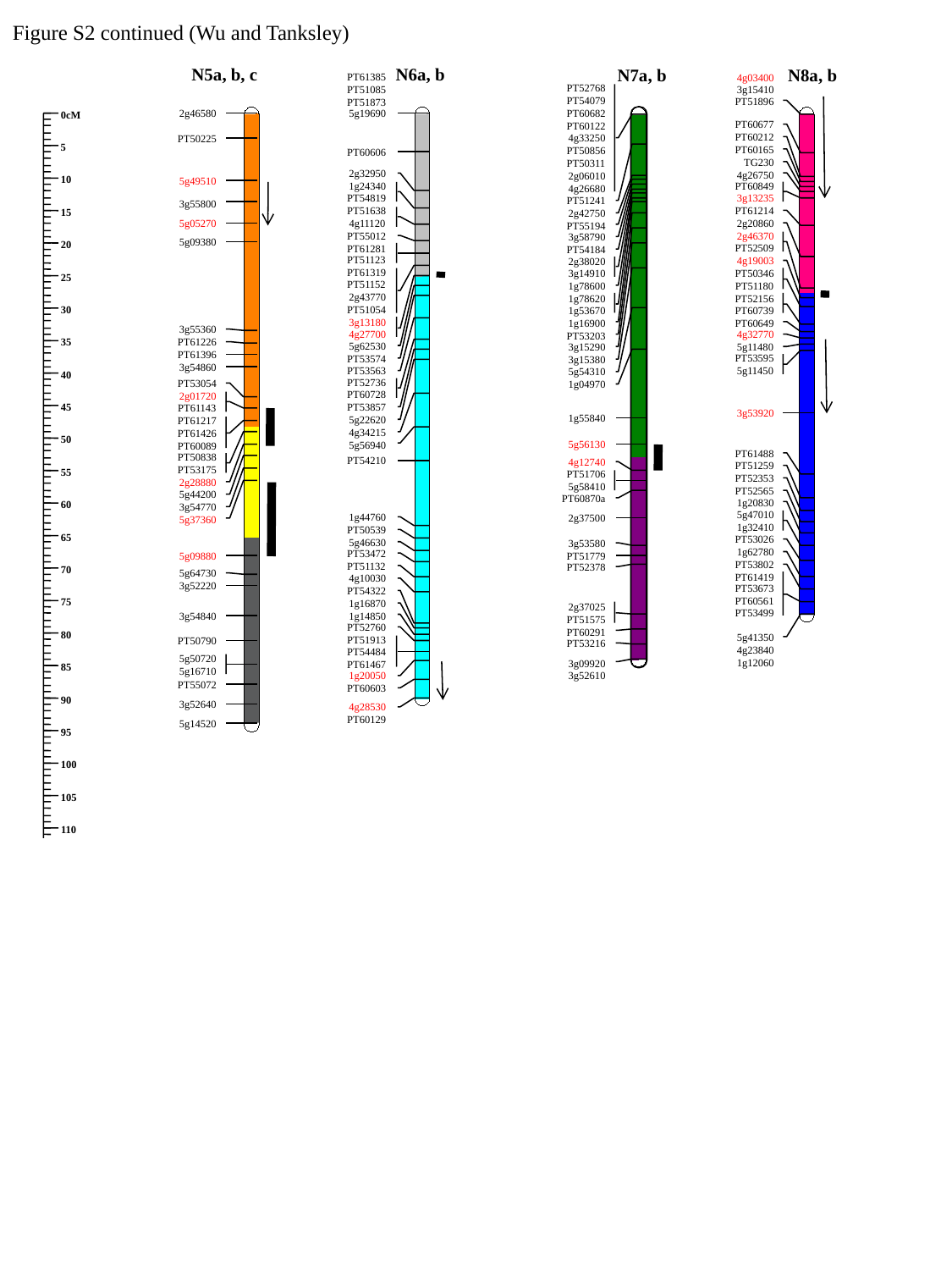

Figure S2 continued (Wu and Tanksley)
N5a, b, c
2g46580
PT50225
5g49510
3g55800
5g05270
5g09380
3g55360
PT61226
PT61396
3g54860
PT53054
2g01720
PT61143
PT61217
PT61426
PT60089
PT50838
PT53175
2g28880
5g44200
3g54770
5g37360
5g09880
5g64730
3g52220
3g54840
PT50790
5g50720
5g16710
PT55072
3g52640
5g14520
N6a, b
PT61385
PT51085
PT51873
5g19690
PT60606
2g32950
1g24340
PT54819
PT51638
4g11120
PT55012
PT61281
PT51123
PT61319
PT51152
2g43770
PT51054
3g13180
4g27700
5g62530
PT53574
PT53563
PT52736
PT60728
PT53857
5g22620
4g34215
5g56940
PT54210
1g44760
PT50539
5g46630
PT53472
PT51132
4g10030
PT54322
1g16870
1g14850
PT52760
PT51913
PT54484
PT61467
1g20050
PT60603
4g28530
PT60129
N7a, b
PT52768
PT54079
PT60682
PT60122
4g33250
PT50856
PT50311
2g06010
4g26680
PT51241
2g42750
PT55194
3g58790
PT54184
2g38020
3g14910
1g78600
1g78620
1g53670
1g16900
PT53203
3g15290
3g15380
5g54310
1g04970
1g55840
5g56130
4g12740
PT51706
5g58410
PT60870a
2g37500
3g53580
PT51779
PT52378
2g37025
PT51575
PT60291
PT53216
3g09920
3g52610
N8a, b
4g03400
3g15410
PT51896
PT60677
PT60212
PT60165
TG230
4g26750
PT60849
3g13235
PT61214
2g20860
2g46370
PT52509
4g19003
PT50346
PT51180
PT52156
PT60739
PT60649
4g32770
5g11480
PT53595
5g11450
3g53920
PT61488
PT51259
PT52353
PT52565
1g20830
5g47010
1g32410
PT53026
1g62780
PT53802
PT61419
PT53673
PT60561
PT53499
5g41350
4g23840
1g12060
0cM
5
10
15
20
25
30
35
40
45
50
55
60
65
70
75
80
85
90
95
100
105
110

## Slide 10
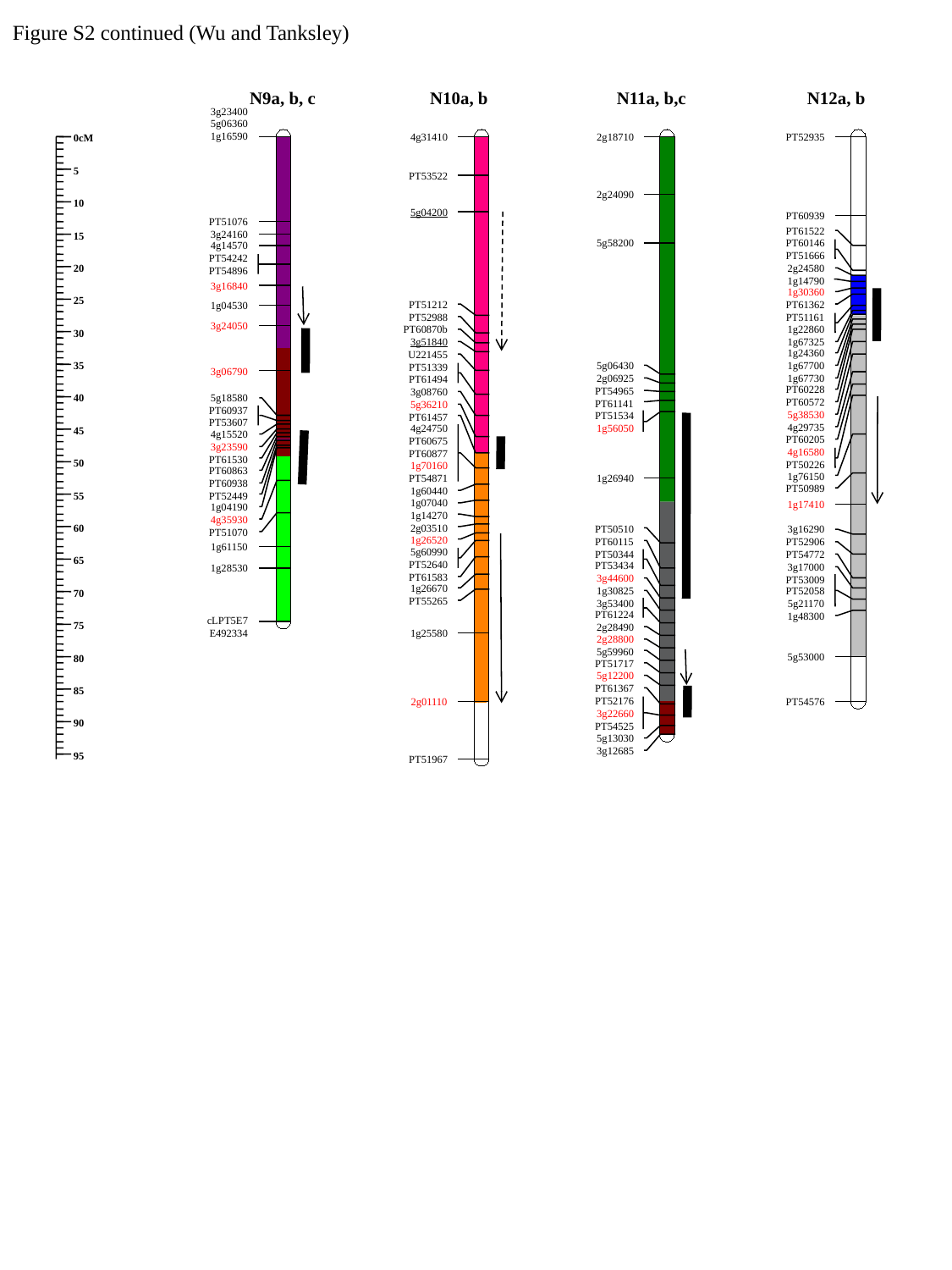

Figure S2 continued (Wu and Tanksley)
N9a, b, c
3g23400
5g06360
1g16590
PT51076
3g24160
4g14570
PT54242
PT54896
3g16840
1g04530
3g24050
3g06790
5g18580
PT60937
PT53607
4g15520
3g23590
PT61530
PT60863
PT60938
PT52449
1g04190
4g35930
PT51070
1g61150
1g28530
cLPT5E7
E492334
N10a, b
4g31410
PT53522
5g04200
PT51212
PT52988
PT60870b
3g51840
U221455
PT51339
PT61494
3g08760
5g36210
PT61457
4g24750
PT60675
PT60877
1g70160
PT54871
1g60440
1g07040
1g14270
2g03510
1g26520
5g60990
PT52640
PT61583
1g26670
PT55265
1g25580
2g01110
PT51967
N11a, b,c
2g18710
2g24090
5g58200
5g06430
2g06925
PT54965
PT61141
PT51534
1g56050
1g26940
PT50510
PT60115
PT50344
PT53434
3g44600
1g30825
3g53400
PT61224
2g28490
2g28800
5g59960
PT51717
5g12200
PT61367
PT52176
3g22660
PT54525
5g13030
3g12685
N12a, b
PT52935
PT60939
PT61522
PT60146
PT51666
2g24580
1g14790
1g30360
PT61362
PT51161
1g22860
1g67325
1g24360
1g67700
1g67730
PT60228
PT60572
5g38530
4g29735
PT60205
4g16580
PT50226
1g76150
PT50989
1g17410
3g16290
PT52906
PT54772
3g17000
PT53009
PT52058
5g21170
1g48300
5g53000
PT54576
0cM
5
10
15
20
25
30
35
40
45
50
55
60
65
70
75
80
85
90
95
