# Supplementary material for: Chromosomal evolution in the plant family Solanaceae
Source: BMC Genomics. 2010 Mar 17;11:182. doi: 10.1186/1471-2164-11-182 (PMC2847972; doi:10.1186/1471-2164-11-182)
Supplement: Additional file 3 — Figure S3 - Comparative maps of several solanaceous species and the deduced genome arrangement of MRCAs (close-up of Figure 4). Designation of chromosome and chromosome segment (a-c) as well as color codes follow Additional File 2. Nomenclature of MRCAs (ATPt, ATE and ATP) follows Figure 1. Maps of non-tomato species are depicted in a comparative way to the tomato map as follows. A black arrow depicts an inversion relative to tomato (a grey arrow for an uncertain inversion). A black bar depicts the breakpoint region of a translocation relative to tomato (a grey bar for an uncertain translocation). Two black bars connected by a curve indicate that the segment in between is excised in a translocation while the remained parts stay together, e.g. E10a is embedded in E3b. "1+" (or "2+") on a single arrow indicates that the region has experienced at least one (or two) inversions but the exact number remains to be determined. Markers displayed on the tomato map were used to define breakpoint regions of translocations and borders of inversions. The prefix (in parentheses) of a marker name specifies at which non-tomato maps the marker locates (E = eggplant, P = pepper, N = Nicotiana). Due to a different tomato map used for tomato-potato comparison, location and length of inversions on the potato map are approximate [3,8]. White dots indicate the approximate centromere location of the tomato chromosomes. [file 1471-2164-11-182-S3.PPT]

## Slide 1
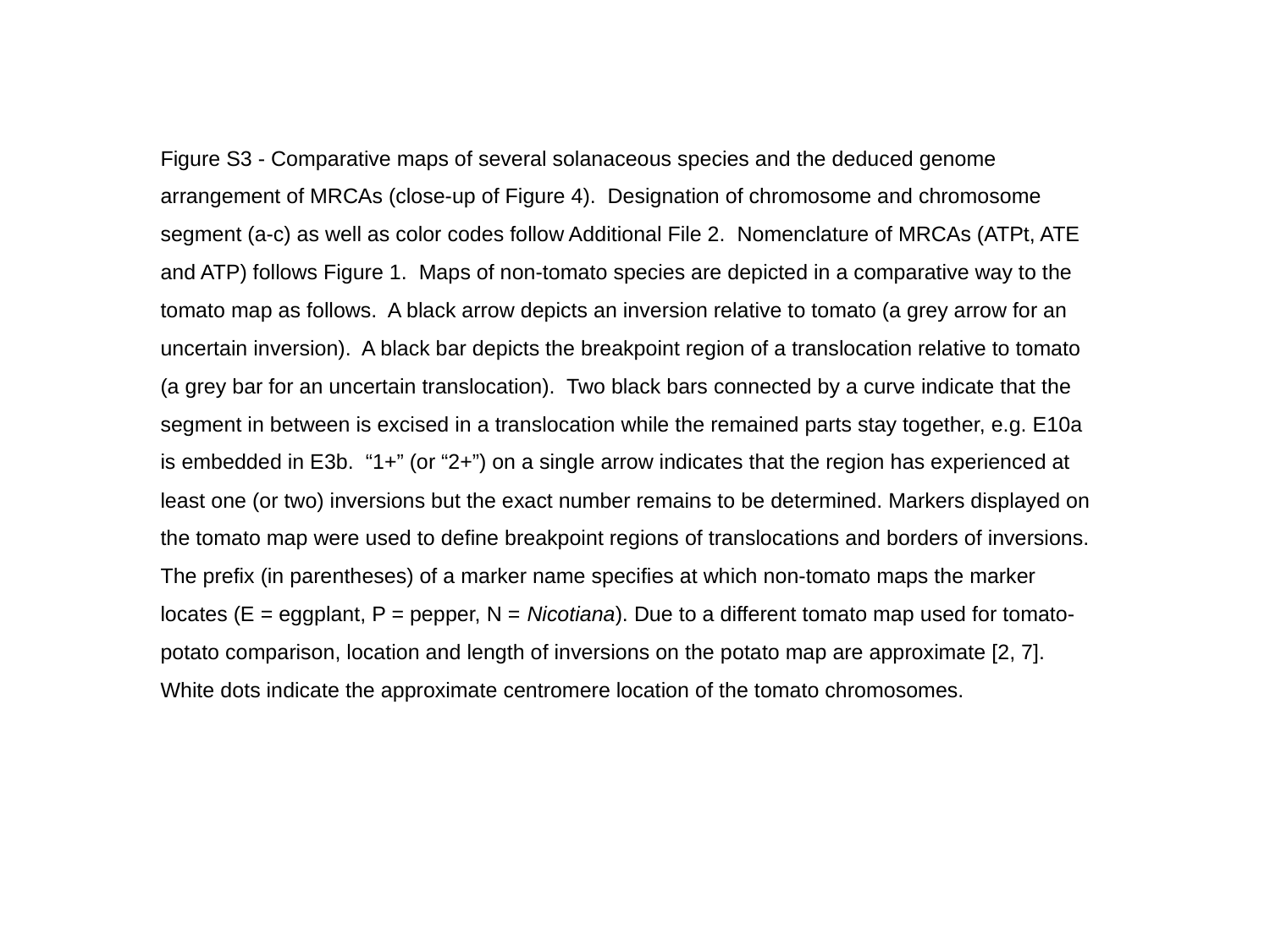

Figure S3 - Comparative maps of several solanaceous species and the deduced genome arrangement of MRCAs (close-up of Figure 4). Designation of chromosome and chromosome segment (a-c) as well as color codes follow Additional File 2. Nomenclature of MRCAs (ATPt, ATE and ATP) follows Figure 1. Maps of non-tomato species are depicted in a comparative way to the tomato map as follows. A black arrow depicts an inversion relative to tomato (a grey arrow for an uncertain inversion). A black bar depicts the breakpoint region of a translocation relative to tomato (a grey bar for an uncertain translocation). Two black bars connected by a curve indicate that the segment in between is excised in a translocation while the remained parts stay together, e.g. E10a is embedded in E3b. “1+” (or “2+”) on a single arrow indicates that the region has experienced at least one (or two) inversions but the exact number remains to be determined. Markers displayed on the tomato map were used to define breakpoint regions of translocations and borders of inversions. The prefix (in parentheses) of a marker name specifies at which non-tomato maps the marker locates (E = eggplant, P = pepper, N = Nicotiana). Due to a different tomato map used for tomato-potato comparison, location and length of inversions on the potato map are approximate [2, 7]. White dots indicate the approximate centromere location of the tomato chromosomes.

## Slide 2
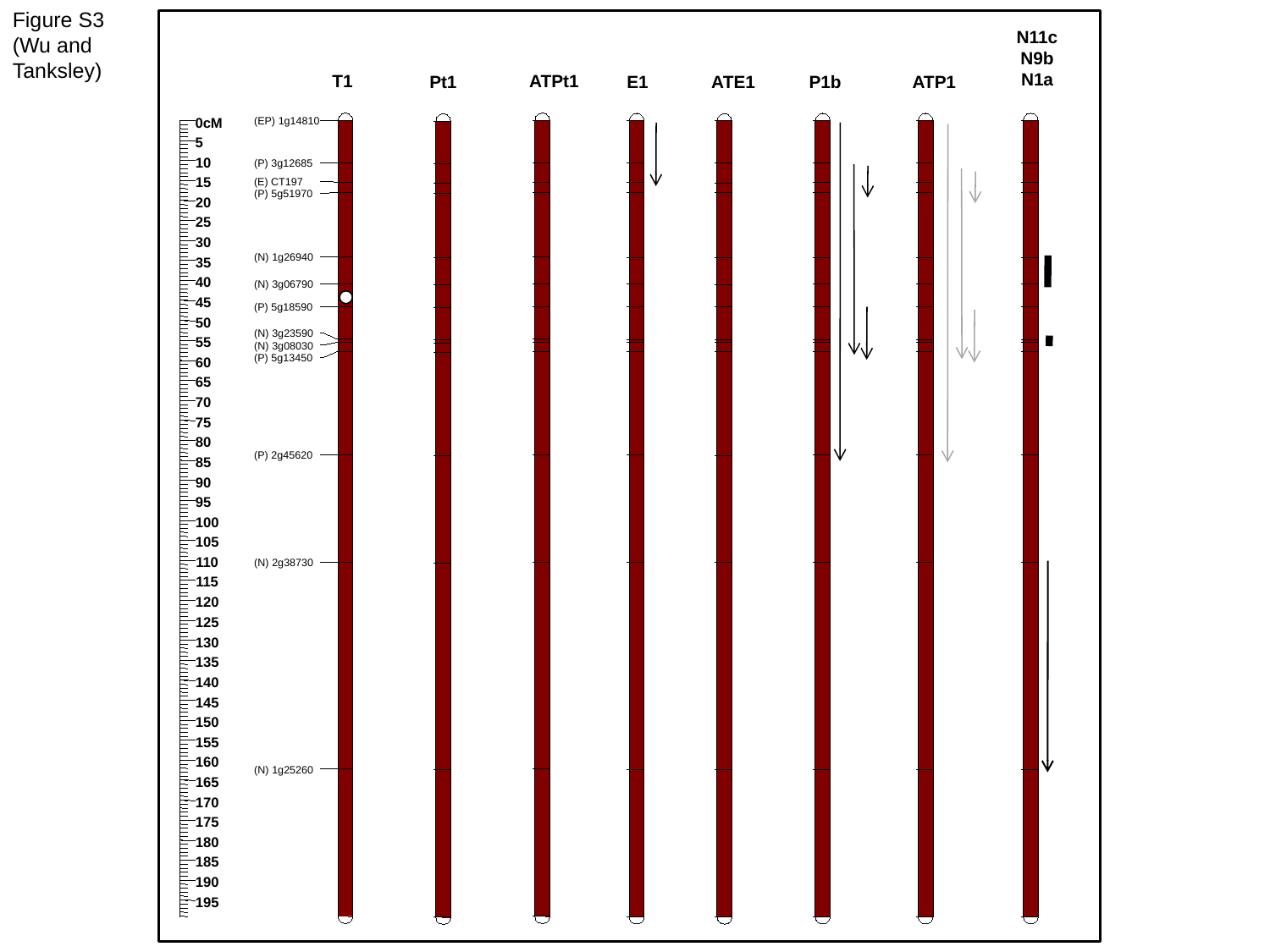

Figure S3
(Wu and Tanksley)
N11c
N9b
N1a
T1
(EP) 1g14810
(P) 3g12685
(E) CT197
(P) 5g51970
(N) 1g26940
(N) 3g06790
(P) 5g18590
(N) 3g23590
(N) 3g08030
(P) 5g13450
(P) 2g45620
(N) 2g38730
(N) 1g25260
ATPt1
E1
P1b
ATP1
ATE1
Pt1
0cM
5
10
15
20
25
30
35
40
45
50
55
60
65
70
75
80
85
90
95
100
105
110
115
120
125
130
135
140
145
150
155
160
165
170
175
180
185
190
195

## Slide 3
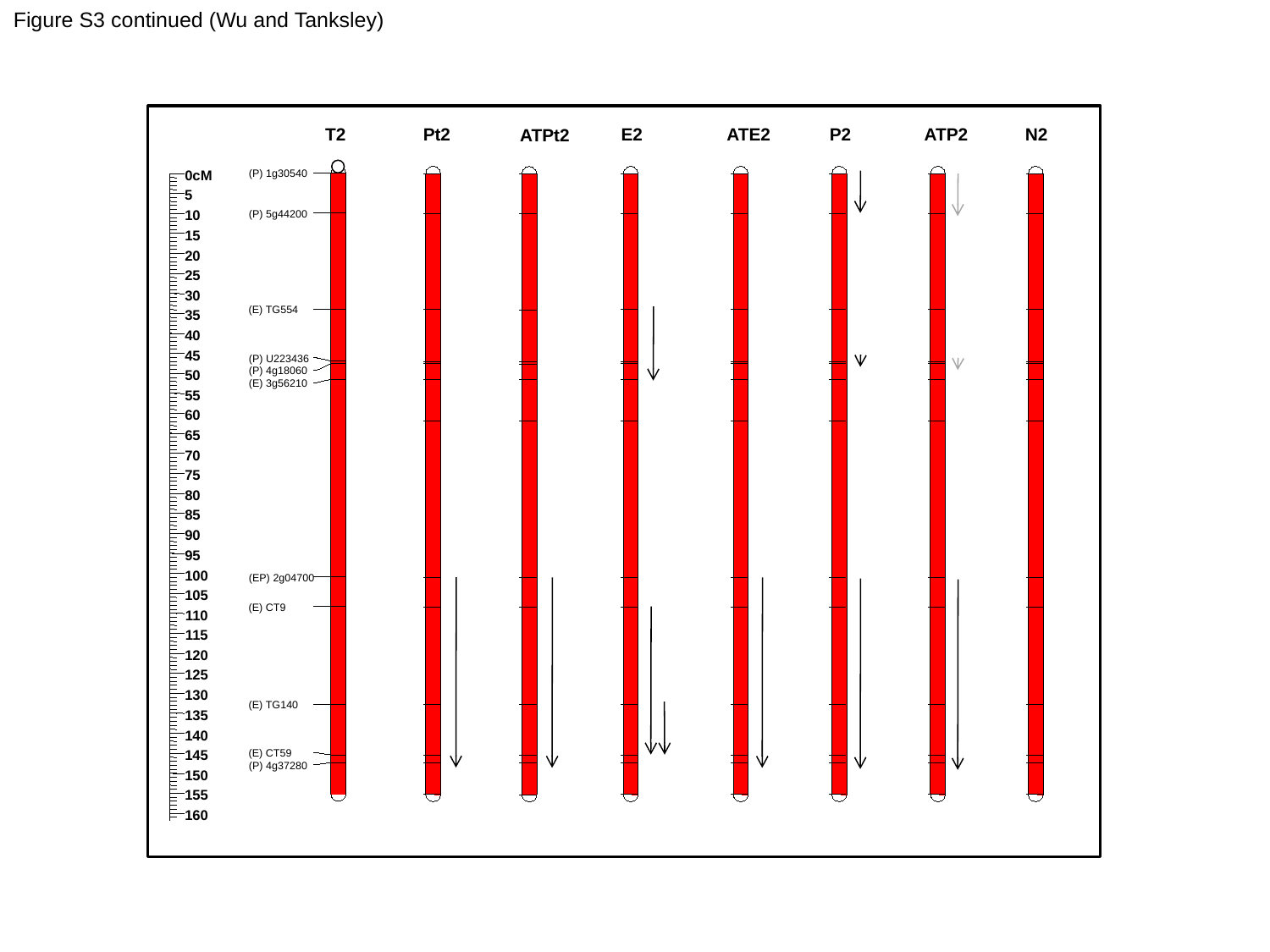

Figure S3 continued (Wu and Tanksley)
T2
(P) 1g30540
(P) 5g44200
(E) TG554
(P) U223436
(P) 4g18060
(E) 3g56210
(EP) 2g04700
(E) CT9
(E) TG140
(E) CT59
(P) 4g37280
Pt2
E2
ATE2
P2
ATP2
N2
ATPt2
0cM
5
10
15
20
25
30
35
40
45
50
55
60
65
70
75
80
85
90
95
100
105
110
115
120
125
130
135
140
145
150
155
160

## Slide 4
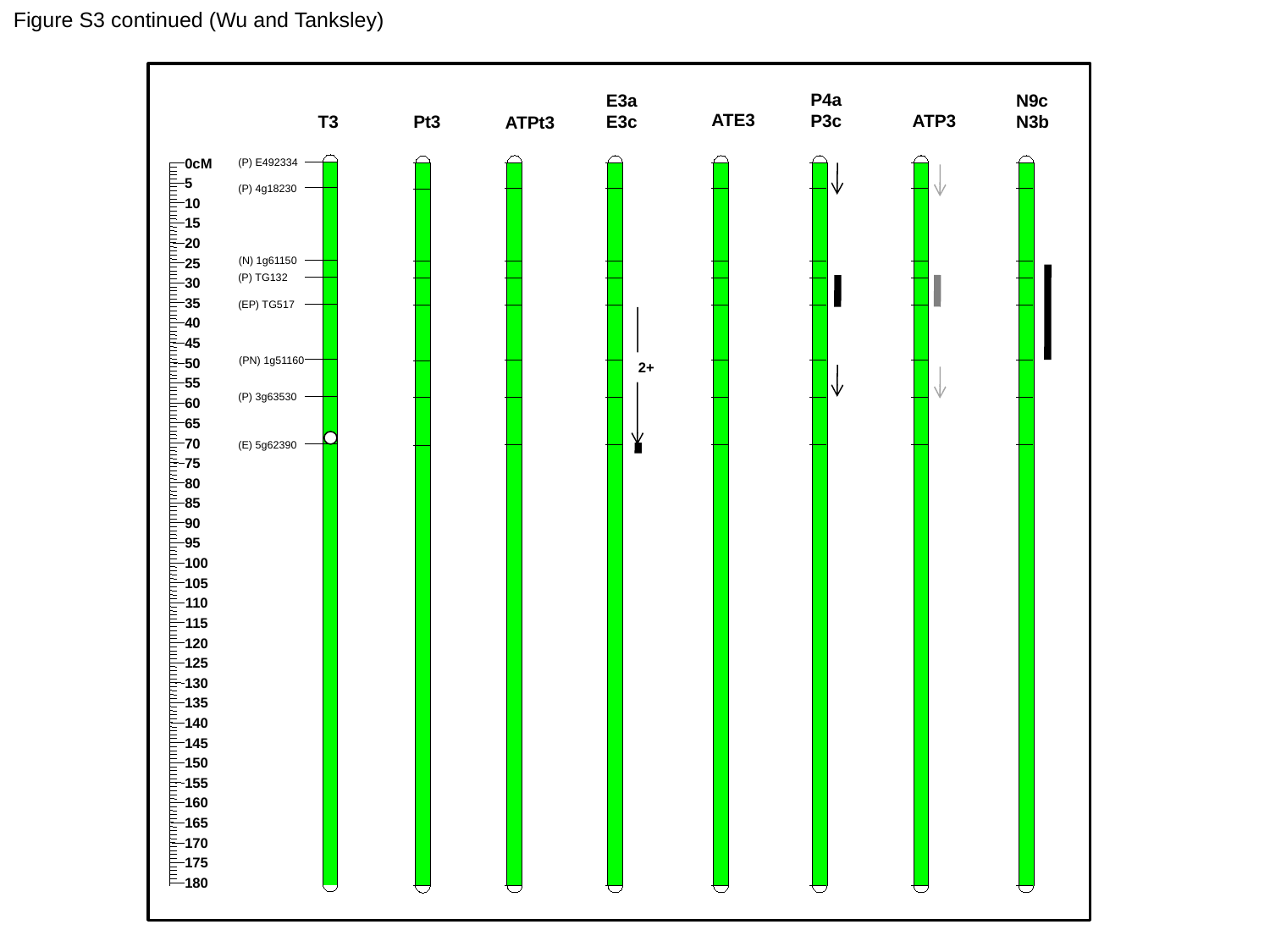

Figure S3 continued (Wu and Tanksley)
P4a
P3c
E3a
E3c
2+
N9c
N3b
ATE3
ATP3
T3
(P) E492334
(P) 4g18230
(N) 1g61150
(P) TG132
(EP) TG517
(PN) 1g51160
(P) 3g63530
(E) 5g62390
Pt3
ATPt3
0cM
5
10
15
20
25
30
35
40
45
50
55
60
65
70
75
80
85
90
95
100
105
110
115
120
125
130
135
140
145
150
155
160
165
170
175
180

## Slide 5
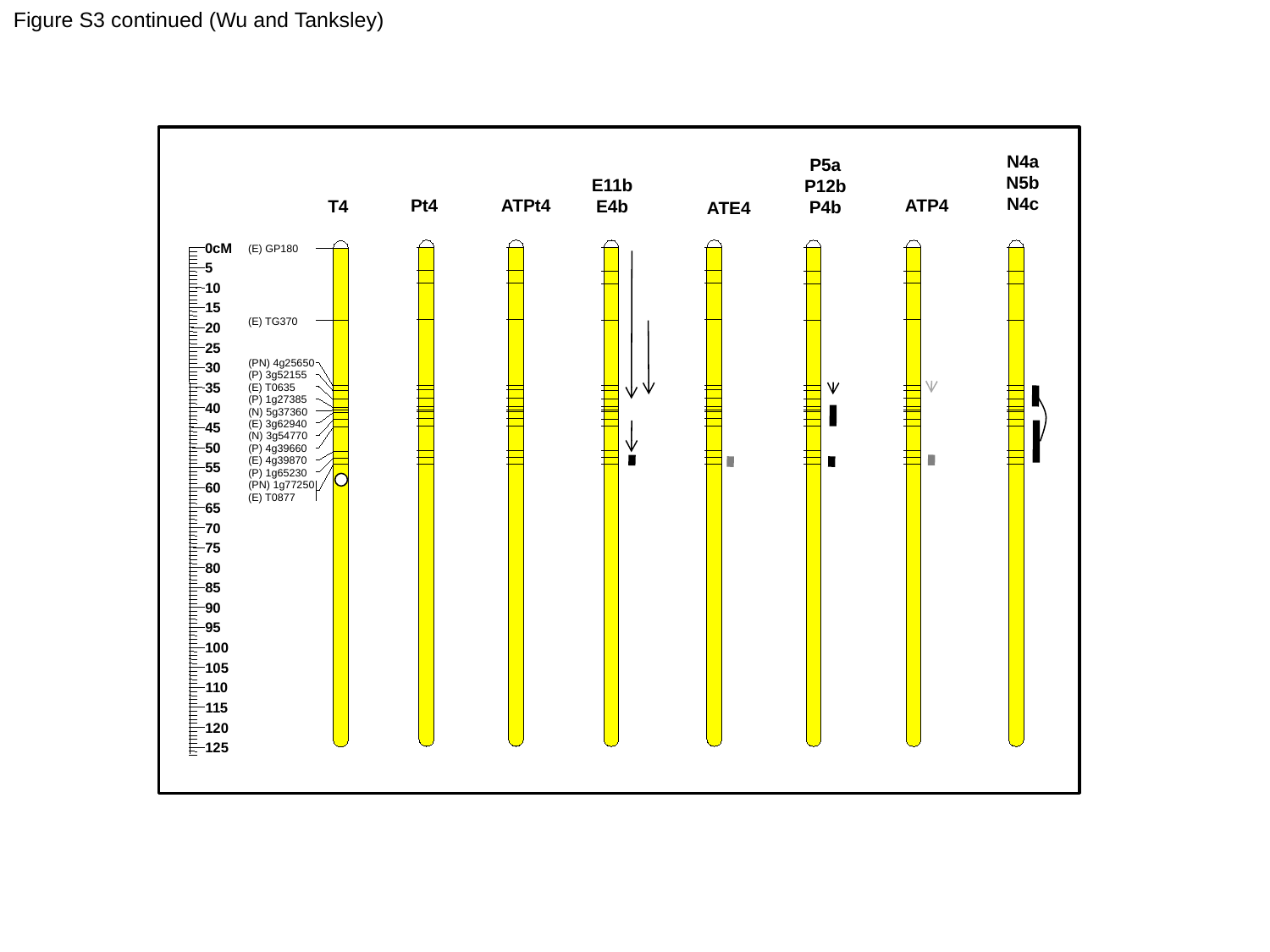

Figure S3 continued (Wu and Tanksley)
N4a
N5b
N4c
P5a
P12b
P4b
E11b
E4b
ATP4
Pt4
ATPt4
T4
(E) GP180
(E) TG370
(PN) 4g25650
(P) 3g52155
(E) T0635
(P) 1g27385
(N) 5g37360
(E) 3g62940
(N) 3g54770
(P) 4g39660
(E) 4g39870
(P) 1g65230
(PN) 1g77250
(E) T0877
ATE4
0cM
5
10
15
20
25
30
35
40
45
50
55
60
65
70
75
80
85
90
95
100
105
110
115
120
125

## Slide 6
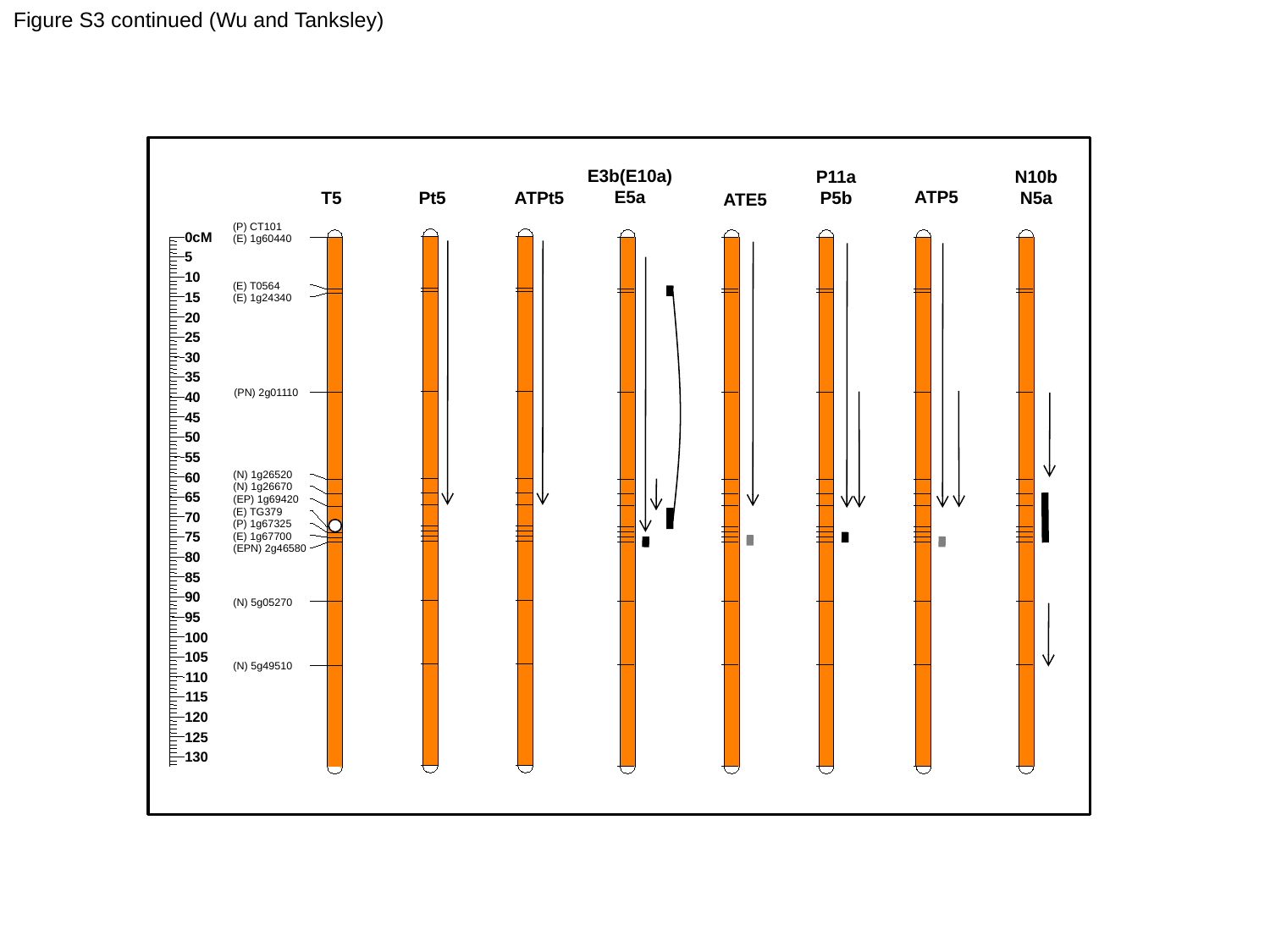

Figure S3 continued (Wu and Tanksley)
E3b(E10a)
E5a
P11a
P5b
N10b
N5a
ATP5
T5
(P) CT101
(E) 1g60440
(E) T0564
(E) 1g24340
(PN) 2g01110
(N) 1g26520
(N) 1g26670
(EP) 1g69420
(E) TG379
(P) 1g67325
(E) 1g67700
(EPN) 2g46580
(N) 5g05270
(N) 5g49510
Pt5
ATPt5
ATE5
0cM
5
10
15
20
25
30
35
40
45
50
55
60
65
70
75
80
85
90
95
100
105
110
115
120
125
130

## Slide 7
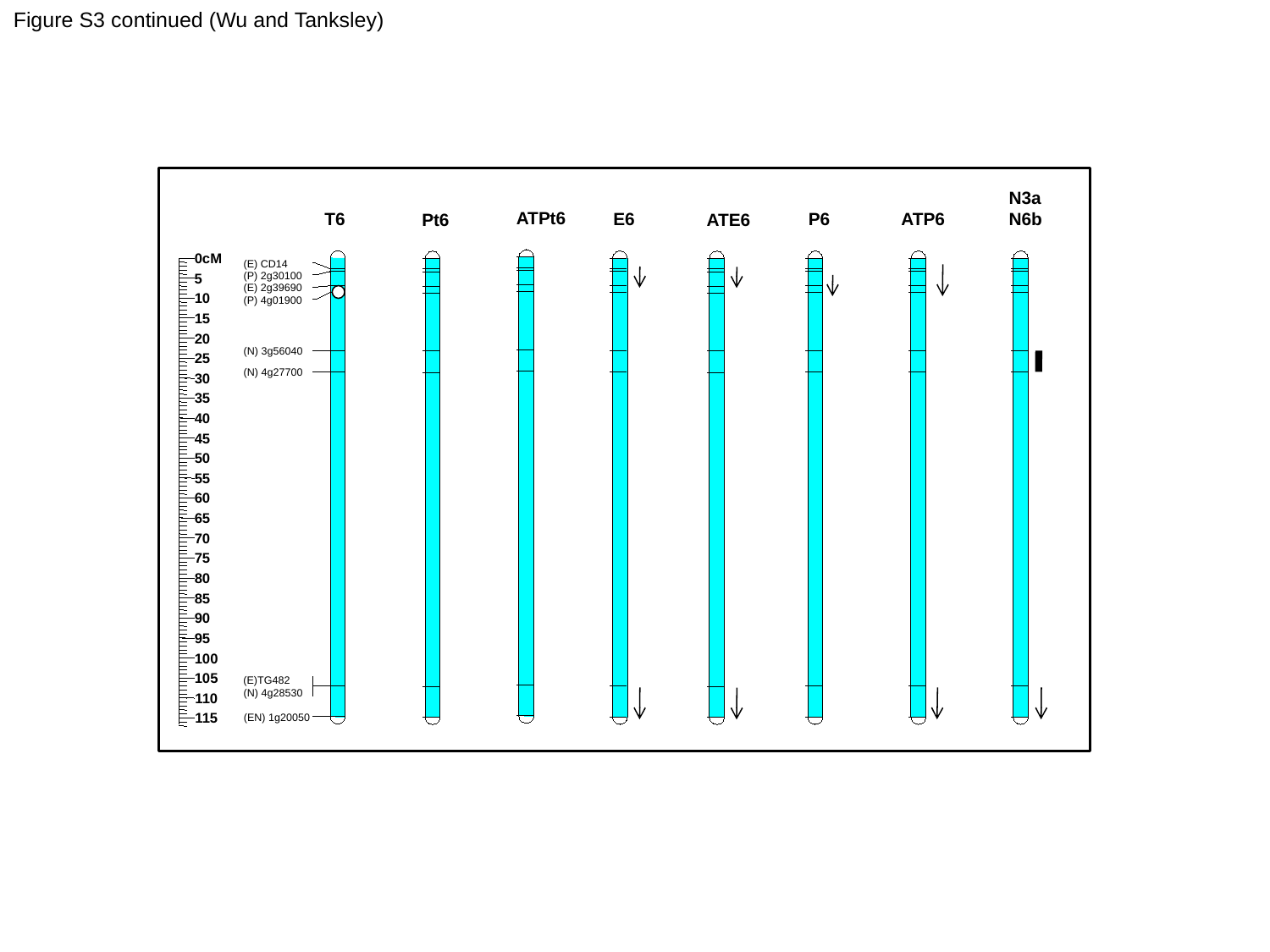

Figure S3 continued (Wu and Tanksley)
N3a
N6b
ATPt6
T6
(E) CD14
(P) 2g30100
(E) 2g39690
(P) 4g01900
(N) 3g56040
(N) 4g27700
(E)TG482
(N) 4g28530
(EN) 1g20050
E6
P6
ATP6
Pt6
ATE6
0cM
5
10
15
20
25
30
35
40
45
50
55
60
65
70
75
80
85
90
95
100
105
110
115

## Slide 8
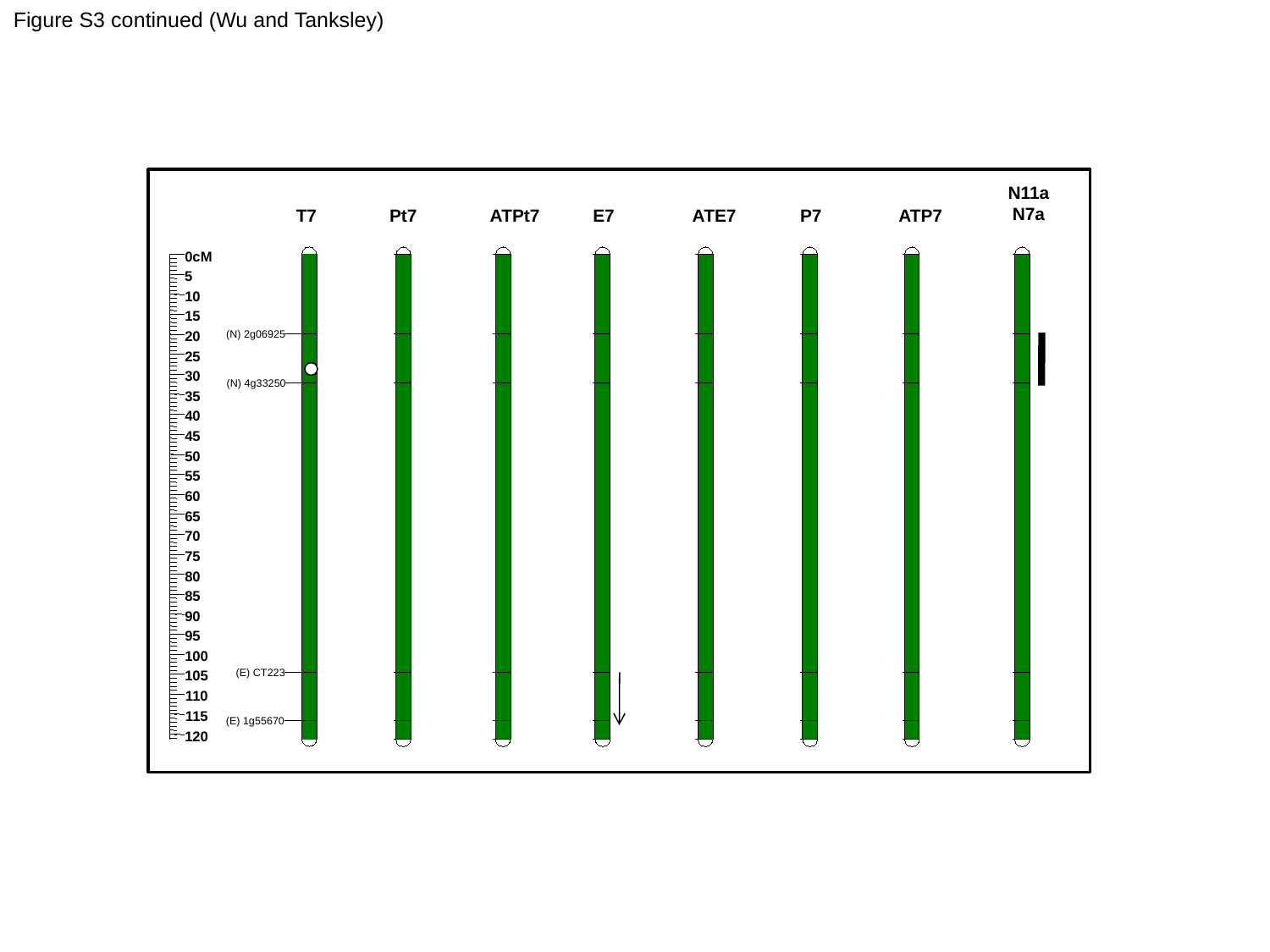

Figure S3 continued (Wu and Tanksley)
N11a
N7a
T7
(N) 2g06925
(N) 4g33250
(E) CT223
(E) 1g55670
Pt7
ATPt7
E7
ATE7
P7
ATP7
0cM
5
10
15
20
25
30
35
40
45
50
55
60
65
70
75
80
85
90
95
100
105
110
115
120

## Slide 9
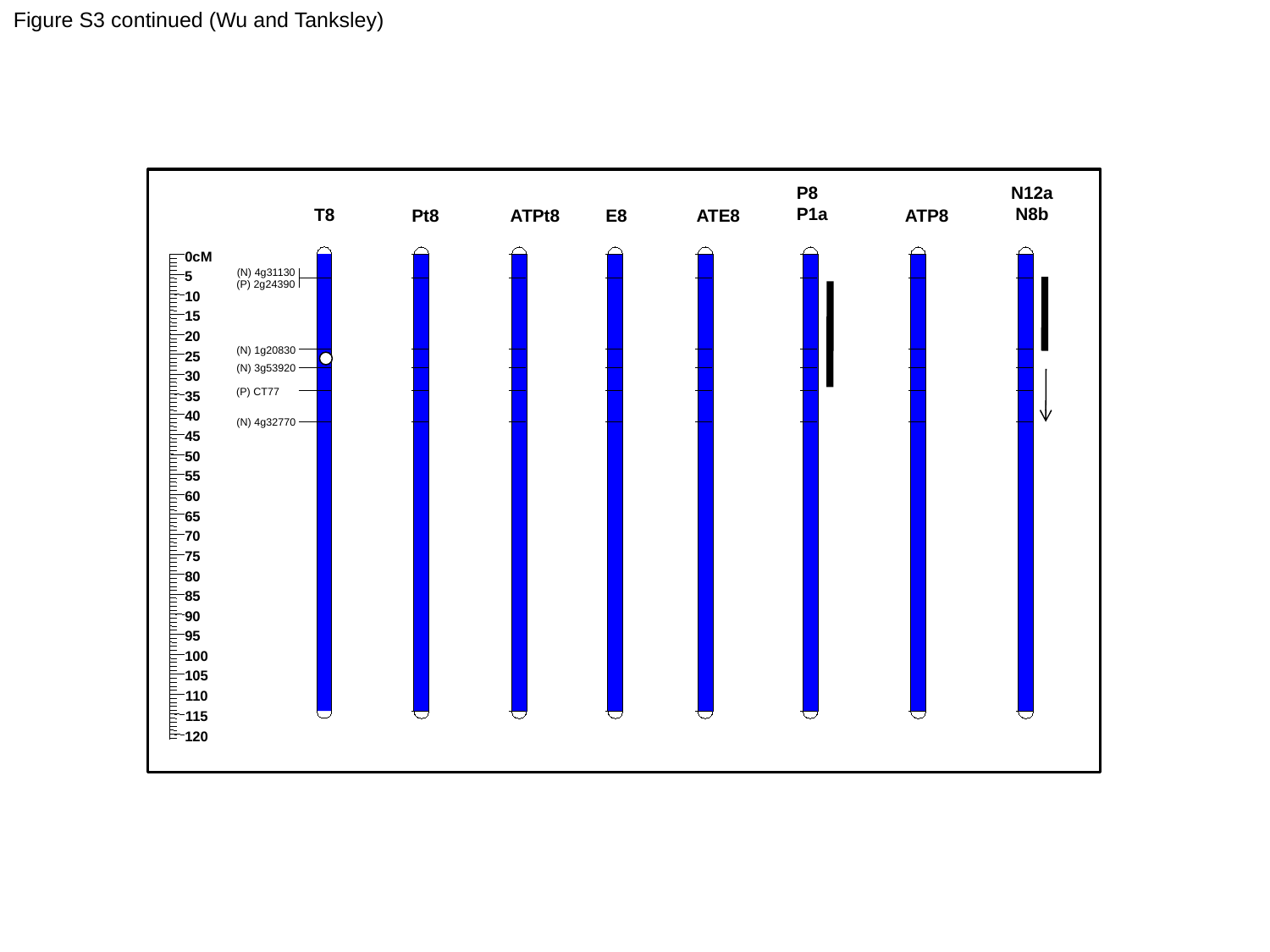

Figure S3 continued (Wu and Tanksley)
P8
P1a
N12a
N8b
T8
(N) 4g31130
(P) 2g24390
(N) 1g20830
(N) 3g53920
(P) CT77
(N) 4g32770
ATP8
Pt8
ATPt8
E8
ATE8
0cM
5
10
15
20
25
30
35
40
45
50
55
60
65
70
75
80
85
90
95
100
105
110
115
120

## Slide 10
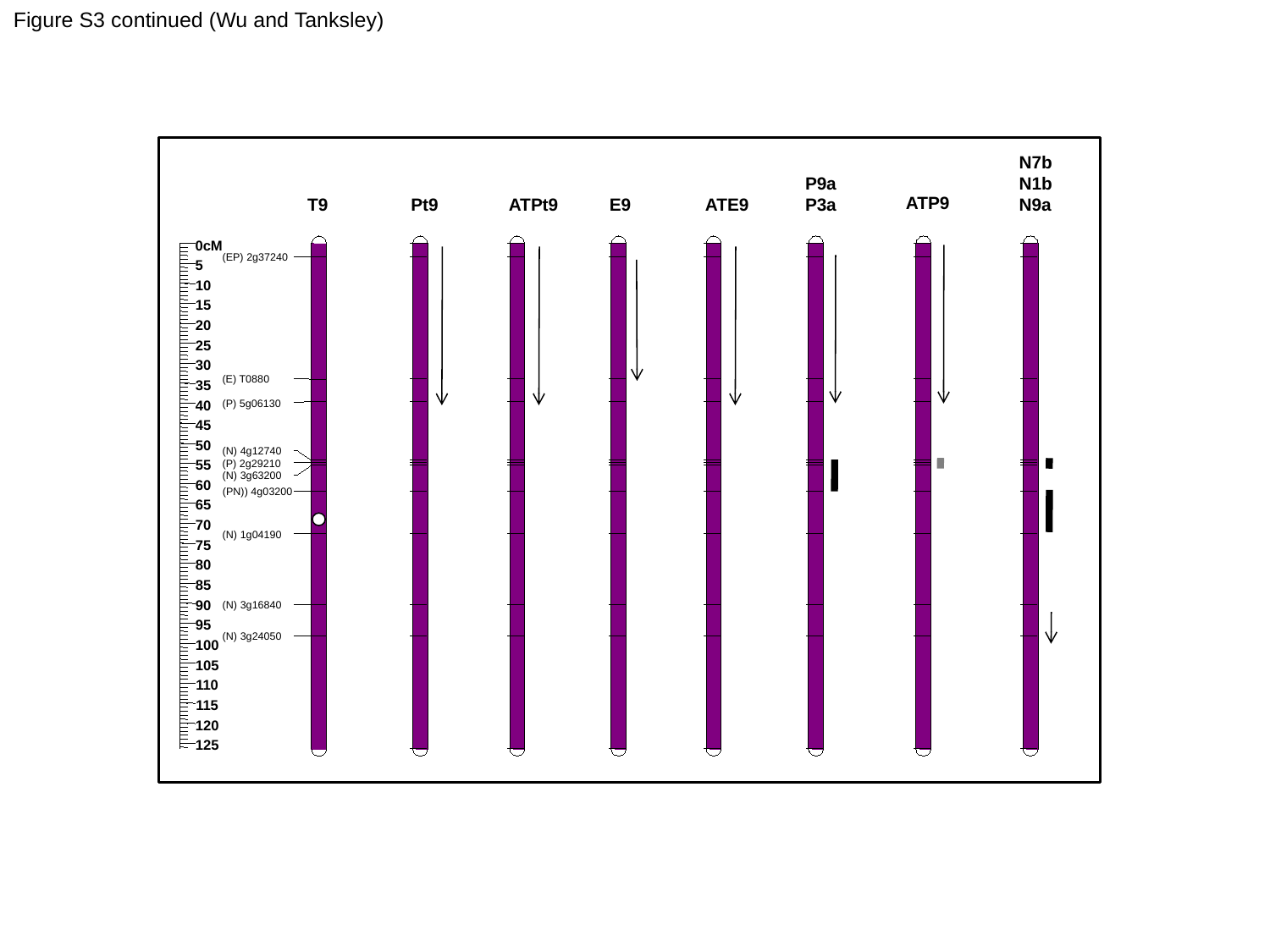

Figure S3 continued (Wu and Tanksley)
N7b
N1b
N9a
P9a
P3a
ATP9
T9
(EP) 2g37240
(E) T0880
(P) 5g06130
(N) 4g12740
(P) 2g29210
(N) 3g63200
(PN)) 4g03200
(N) 1g04190
(N) 3g16840
(N) 3g24050
Pt9
ATPt9
E9
ATE9
0cM
5
10
15
20
25
30
35
40
45
50
55
60
65
70
75
80
85
90
95
100
105
110
115
120
125

## Slide 11
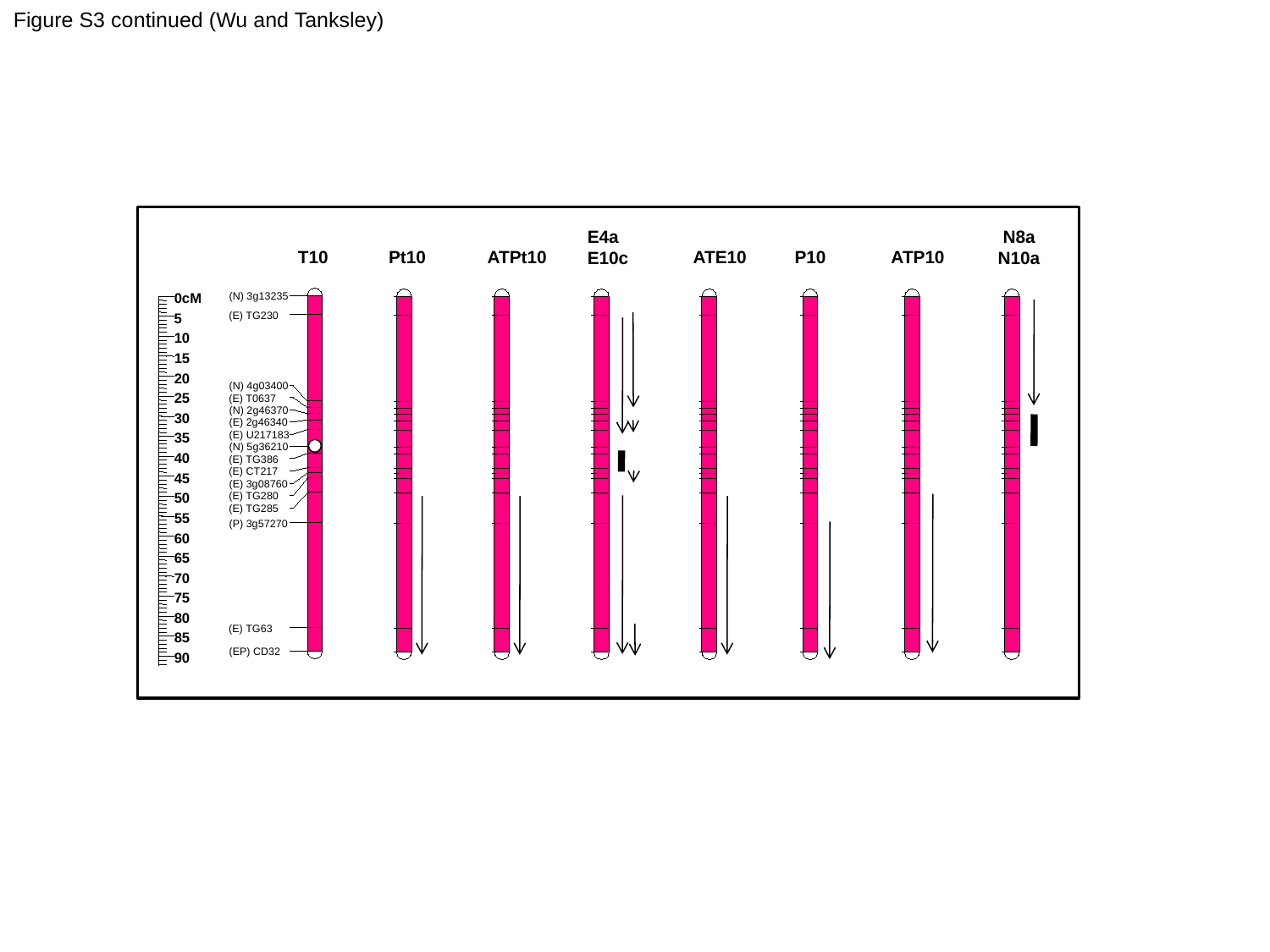

Figure S3 continued (Wu and Tanksley)
E4a
E10c
N8a
N10a
T10
(N) 3g13235
(E) TG230
(N) 4g03400
(E) T0637
(N) 2g46370
(E) 2g46340
(E) U217183
(N) 5g36210
(E) TG386
(E) CT217
(E) 3g08760
(E) TG280
(E) TG285
(P) 3g57270
(E) TG63
(EP) CD32
Pt10
ATPt10
ATE10
P10
ATP10
0cM
5
10
15
20
25
30
35
40
45
50
55
60
65
70
75
80
85
90

## Slide 12
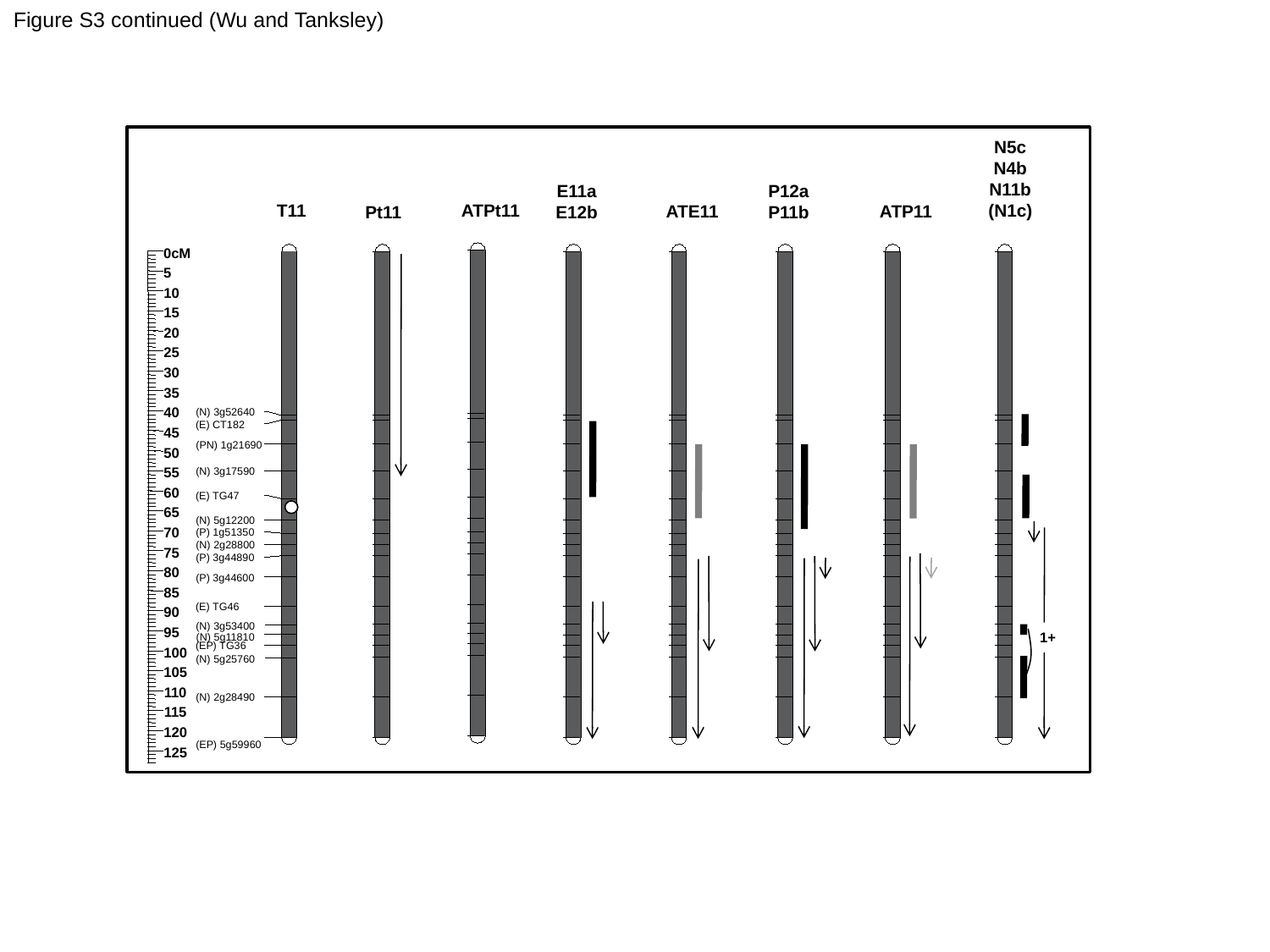

Figure S3 continued (Wu and Tanksley)
N5c
N4b
N11b
(N1c)
1+
E11a
E12b
P12a
P11b
T11
(N) 3g52640
(E) CT182
(PN) 1g21690
(N) 3g17590
(E) TG47
(N) 5g12200
(P) 1g51350
(N) 2g28800
(P) 3g44890
(P) 3g44600
(E) TG46
(N) 3g53400
(N) 5g11810
(EP) TG36
(N) 5g25760
(N) 2g28490
(EP) 5g59960
ATPt11
ATE11
ATP11
Pt11
0cM
5
10
15
20
25
30
35
40
45
50
55
60
65
70
75
80
85
90
95
100
105
110
115
120
125

## Slide 13
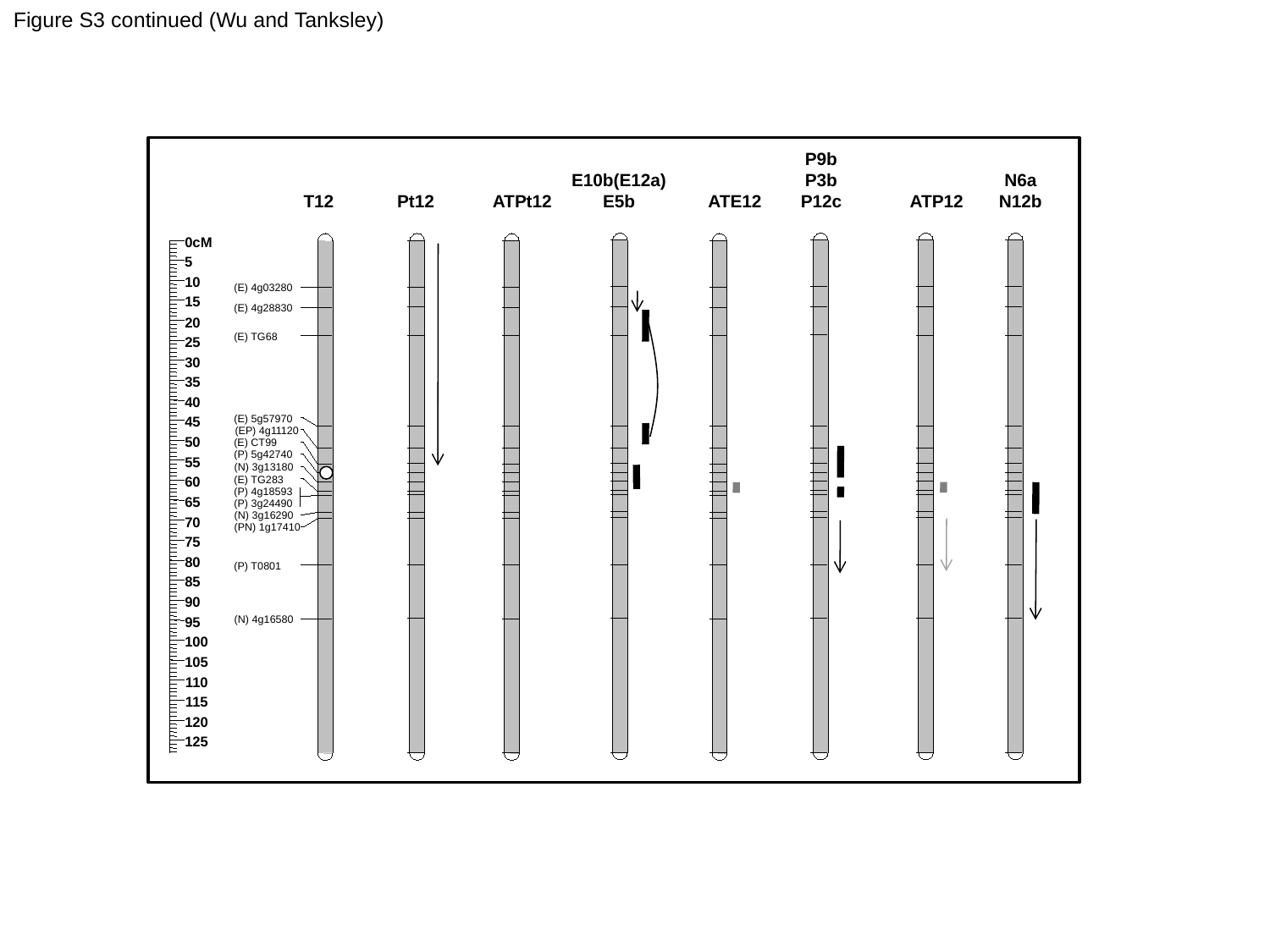

Figure S3 continued (Wu and Tanksley)
P9b
P3b
P12c
E10b(E12a)
E5b
N6a
N12b
T12
(E) 4g03280
(E) 4g28830
(E) TG68
(E) 5g57970
(EP) 4g11120
(E) CT99
(P) 5g42740
(N) 3g13180
(E) TG283
(P) 4g18593
(P) 3g24490
(N) 3g16290
(PN) 1g17410
(P) T0801
(N) 4g16580
Pt12
ATE12
ATP12
ATPt12
0cM
5
10
15
20
25
30
35
40
45
50
55
60
65
70
75
80
85
90
95
100
105
110
115
120
125
